# Redistribution and beliefs about the source of income inequality

**DOI:** 10.1007/s10683-021-09733-8

**Published:** 2021-09-27

**Authors:** Vanessa Valero

**Affiliations:** 1 Loughborough University, Loughborough, UK; 2 CeDEx, Nottingham, UK

**Keywords:** Redistribution, Fairness, (Motivated) Beliefs, Laboratory experiments, D31, D63, D64, D83, H23

## Abstract

Previous literature demonstrates that beliefs about the determinants of income inequality play a major role in individual support for income redistribution. This study investigates how people form beliefs regarding the extent to which work versus luck determines income inequality. Specifically, I examine whether people form self-serving beliefs to justify supporting personally advantageous redistributive policies. I use a laboratory experiment where I directly measure beliefs and manipulate the incentives to engage in self-deception. I first replicate earlier results demonstrating that (1) people attribute income inequality to work when they receive a high income and to luck when they receive a low income and (2) their beliefs about the source of income inequality influence their preferences over redistributive policies. However, I do not find that people’s beliefs about the causes of income inequality are further influenced by self-serving motivations based on a desire to justify favorable redistributive policies. I conclude that, in my experiment, self-serving beliefs about the causes of income inequality are driven primarily by overconfidence and self-image concerns and not to justify favorable redistributive policies.

**Supplementary Information:**

The online version contains supplementary material available at 10.1007/s10683-021-09733-8.

## Introduction

Income inequality has increased almost everywhere over the past few decades (World Inequality Report, 2018). Rising inequality is a concern, as it is negatively associated with long-term growth and creates social problems (e.g., Piketty, 2014; Stiglitz, 2012). Where inequality is a concern, redistributive policies constitute a widely utilized tool for obtaining greater economic equality. An important determinant of the support for these policies is perceptions of deservingness. People are more inclined to support redistribution when they believe that income is due to circumstances beyond individual control (such as luck) rather than within individual control (such as work). This relationship has been documented in both survey data (Fong, 2001; Alesina & Angeletos, 2005, Alesina & La Ferrara, 2005 and Alesina & Giuliano, 2011) and experimental data (e.g., Cappelen et al., 2007, 2013; Fong, 2007).

While it is well known that beliefs about the causes underlying income inequality influence the demand for redistribution, much less is known about how people form these beliefs. This paper investigates whether beliefs about the causes of inequality are, in turn, influenced by the demand for redistribution. Specifically, I investigate whether adopting favorable beliefs about the causes of income inequality provides individuals with a means to justify supporting personally favorable redistributive policies. For example, do the rich exaggerate the extent to which hard work yields success to morally justify not supporting redistribution? Because such causes are often difficult to assess, people must form judgments regarding the role that factors such as hard work and luck played in determining individuals’ success or failure. This offers an opportunity to engage in self-serving belief manipulation, whereby individuals adopt the beliefs that are most convenient and provide the greatest opportunity for favorable level of redistribution. According to standard economic theory, beliefs arise independently of the individuals’ self-interest. However, considerable evidence indicates that beliefs are shaped by self-interest in other settings, such that individuals can both act egoistically while feeling moral about their behavior (see, Gino et al., 2016 for a review and the review of literature in Sect. 2). For this reason, I introduce the possibility that beliefs about the causes underlying economic inequality are susceptible to self-serving biases known to influence belief formation in other settings. Consistent with this possibility, Piketty (2020, page 2) argues that: “the discourse of meritocracy and entrepreneurship often seems to serve primarily as a way for the winners in today’s economy to justify any level of inequality whatsoever while peremptorily blaming the losers for lacking talent, virtue and diligence.”^1^

Previous literature demonstrates that people form self-serving beliefs about the causes underlying success or failure—they systematically attribute their success to circumstances within their control and failure to circumstances beyond their control. This tendency to feel responsible for success but not for failure is usually interpreted by psychological studies as overconfidence, to protect or enhance self-esteem (e.g., Miller & Ross, 1975; Weiner, 1985). However, there is no evidence on whether people also bias their perceptions of the roles of luck and effort in determining outcomes in order to justify adopting personally favorable redistributive policies.

Understanding how people form judgments about deservingness is critical for understanding the extent to which they will support redistribution or how they will react to its implementation. Identifying a potential self-serving bias in beliefs is also important as it can contribute to polarization in attitudes towards redistribution. On the one hand, self-serving biases can reinforce the perception of deservingness of the wealthy, who can subsequently exert their political power to change the rules to make inequality more persistent. On the other hand, if the poor overestimate the role played by luck in determining income, they will reject inequality even more and pursue redistributive policies to a larger extent. A political consensus about redistribution might then be more difficult to obtain, increasing political tensions and class conflicts.

I start by studying correlational evidence between attitudes toward redistribution and beliefs about the source of income using the World Values Survey. The results indicate that people who are less inclined to support redistribution are also more likely to believe that income is due mainly to work. This result is consistent with earlier findings that beliefs about the sources of income influence support for redistribution, but also with the possibility that attitudes towards redistribution affect the beliefs about what determine economic success or failure. The observational data therefore cannot provide any causal evidence of a relationship between a desire for redistribution and beliefs about the sources of income, and thus cannot provide any evidence of belief distortion. In this paper I investigate this relationship using a laboratory experiment, where it is possible to both directly measure beliefs and manipulate the incentives potentially underlying their formation. To the best of my knowledge, this is the first study investigating the causal effect of preferences for redistribution on the beliefs about the determinants of income inequality.

In the experiment, participants perform a work task after which they receive either a high or low level of initial income. Income is determined either according to participants’ relative work performance or luck. Participants are aware of these two possibilities, but do not know whether, in their case, income was actually determined by work performance or luck.^2^ I then elicit beliefs, in an incentive-compatible manner, regarding the role of work versus luck. If individuals are overconfident or motivated to maintain a positive self-image, their beliefs will demonstrate a self-serving bias: high-income individuals will systematically believe that work performance was more likely to be responsible for income determination than luck, while low-income individuals will believe the opposite.

The main focus of this experiment is on whether a desire to support favorable redistributive policies further impacts beliefs. The treatment variation consists of manipulating whether beliefs are measured *before* or *after* informing participants that they will subsequently decide how much to redistribute from participants with high income to those with low income. In the latter case, high-income participants know that they may be financially harmed by redistribution at the time they form their beliefs. This offers them an opportunity to convince themselves that income inequality is more likely due to work than luck in order to justify redistributing less afterwards. In the former case, they do not know that they will have the possibility to redistribute afterwards, and thus do not have such an incentive to bias their beliefs.^3^ This provides a clean test of whether beliefs reflect a self-serving bias when they are formed with knowledge that redistribution will subsequently occur. I also measure preferences over redistribution, as a way of testing whether these are influenced by the elicited beliefs. Focusing on high-income participants’ redistribution decisions allows me to directly address the question of whether the winners in today’s economy use the discourse of deservingness to justify income inequality and redistributing less, as proposed by Piketty (2020).^4^

My experiment yields several insights. First, the results show that high-income participants believe that they have greater responsibility for their income than low-income participants. Specifically, participants attribute income inequality to a larger extent to their work performance when they receive a high income, and to a larger extent to luck when they receive a low income. This is consistent with the formation of beliefs in a manner that promotes a positive self-image, based on the idea that one’s success is due to factors within personal control and overconfidence regarding one’s relative ability. I also find that beliefs about what causes inequality influence how much participants redistribute. Specifically, people who believe that outcomes were determined by luck favor more redistribution than those who believe it is due to work. Both of these results are consistent with previous findings that I describe in more detail in the next section.

However, I find no evidence that preferences over redistribution further influence the self-serving nature of beliefs. Specifically, participants who receive a high income do not overestimate the importance of work to a greater extent when they know that they will subsequently have the opportunity to engage in redistribution than when they do not know of such an opportunity. Instead, my results demonstrate a precisely estimated null effect of knowledge of the upcoming redistribution possibility on beliefs.

My results thus imply that participants form self-serving beliefs about how much control they have over their outcomes mainly due to overconfidence or a desire to maintain a positive self-image, which is present both when they do and do not have knowledge of subsequent redistribution. Knowing that redistribution decisions are forthcoming does not strengthen the relationship between personal circumstances and beliefs. The role of financial self-interest when forming beliefs about the source of income inequality seems to be minor in my experiment.^5^ Therefore, any observed polarization of beliefs about the causes underlying inequality between the rich and poor within societies should be attributed to alternative motivations, including overconfidence and self-image concerns, and not to a desire to justify self-serving policies.^6^

My paper proceeds as follows. The next section briefly reviews some related work. Section 3 reports the analysis of survey data on the relationship between attitudes toward redistribution and beliefs about the source of income. Section 4 describes the experimental design, and Sect. 5 the hypothesis. Section 6 presents the results while Sect. 7 concludes.

## Related literature

Previous experimental literature provides ample evidence that the source of income inequality influences the decision whether to support income redistribution. Specifically, people redistribute less when income is earned (e.g., through work) rather than determined by luck. This has been shown using dictator games in which an individual can unilaterally decide how much to give to another person (e.g., Cappelen et al., 2007, 2013; Cherry et al., 2002; Hoffman et al., 1994; Rey-Biel et al., 2018). This is also the case when people collectively decide—e.g., through voting—on a redistributive policy (Durante et al., 2014; Krawczyk, 2010; Lefgren et al., 2016).

However, what determines income inequality is usually difficult to assess in real-world contexts. This means that individuals must form judgments regarding the underlying causes of economic success or failure. We know that these beliefs also play an important role in the demand for redistribution. In survey data, those who believe that economic outcomes are due to factors beyond individual control, such as luck, are more likely to support redistribution (Fong, 2001; Alesina & Angeletos, 2005; Alesina & La Ferrara, 2005; Alesina & Giuliano, 2011). Experimental studies have also documented such relationships (Fong, 2007; Fong & Luttmer, 2011).^7^These studies manipulate the perceived deservingness of welfare recipients and confirm that these perceptions are an important determinant of the decision to share wealth with them. Indeed, participants are more likely to give when recipients are believed to be poor because of bad luck rather than lack of effort.^8^

While it is a robust finding that beliefs about the source of income are important for redistribution, very few studies investigate how people form these beliefs and the degree to which self-serving biases influence their formation. Cassar and Klein (2019) find that experience of economic failure or success shapes people’s preference for redistribution, notably due to self-serving bias in responsibility attribution. This bias has been documented by psychological studies (e.g., Miller & Ross, 1975; Weiner, 1985 and Mezulis et al., 2004) that show that people tend to attribute their failure to circumstances beyond individuals’ control, and their success to circumstances within their control such as ability. Some economic studies also provide evidence for self-serving belief manipulation about one’s abilities—i.e., overconfidence (e.g., Möbius, et al., 2017; Coutts et al., 2019; Zimmerman, 2020). In the context of redistribution, Deffains et al. (2016) found that participants in a laboratory experiment who succeeded at a task in which one’s relative performance was determined entirely by luck—i.e., whether one was randomly assigned to perform either a hard or an easy task—are more likely to attribute their success to factors within individual control and exhibit opposition to redistribution afterwards.^9^ They also show that participants’ beliefs about the source of their income influence their preferences for redistribution. This is consistent with the idea that people develop self-serving notions of deservingness, but they do so because they are overestimate of their skills and abilities in the previous papers. No evidence exists that documents whether people distort their perception of deservingness to justify adopting advantageous redistributive policies, and for their own financial self-interest.

At the same time, a large body of research demonstrates that people engage in self-serving belief manipulation to create justifications for behaving egoistically (see Gino et al., 2016, for a review). For instance, individuals engage in self-deception as a justification for egoism in contexts such as dictator games (Dana et al., 2007; Haisley & Weber, 2010), strategic interactions (Di Tella et al, 2015) and charitable donations (Exley, 2016). More closely related to this study, Konow (2000) and Rodriguez-Lara and Moreno-Garrido (2012) demonstrate that people distort their beliefs of what constitutes a fair redistribution in the direction of their own financial self-interest, though they do not explore the role of beliefs about the causes of success or failure. This paper contributes to this literature by examining whether perceptions of deservingness are also susceptible to be formed self-servingly to justify acting in a selfish manner.

This paper is also related to the literature that have recently started to call into question the generalizability of the evidence supporting motivated beliefs to justify acting egoistically. For instance, Van der Weele et al. (2014) show that people do not use excuses to behave unfairly in the context of reciprocity. Bartling and Özdemir (2017) find that people do not use the replacement excuse “if I don’t do it, someone else will” if a social norm of moral behavior exists. Ging-Jehli et al. (2020) do not find evidence that player adopt negative beliefs about others’ intentions to justify egoistic behavior. In this paper, I contribute to this literature by providing one important example, in the context of redistribution, in which people with a financial incentive to engage in belief manipulation do not do so. This is important as it could avoid producing a literature bias leading us to believe that people systematically form self-servingly their beliefs to justify acting in a selfish manner.

## Observational data

This section examines the relationship between attitudes toward redistribution and beliefs about the sources of income using the World Values Survey. In Table 1, I regress individuals’ belief that hard work brings success against their political orientation used as a proxy for the demand of redistribution.Similarly to Alesina and Angeletos (2005), if an individual identifies himself as being on the left of the political spectrum, I take it as a proxy for favoring redistribution. The first column reveals a negative and significant relationship between both variables. This suggests that the less people support redistribution, the more they think income is due to hard work. This is also true if I control for income (second column).^10^ The coefficient for income is positive and statistically significant, indicating that the richer individuals are, the more they think income is due to hard work.^11^ The third column introduces individual characteristics and countries as explanatory variables and confirms previous findings. The significant and robust negative relationship between redistribution and beliefs about the sources of income is consistent with earlier findings that beliefs about the sources of income influence the support for redistribution, but also with the idea that beliefs are formed self-servingly to justify supporting personally advantageous redistributive policies. Because these data only provide *correlational* evidence, I cannot impute causality.

**Table 1:** OLS regressions of individuals’ belief that hard work brings success

	(1)	(2)	(3)
*Support for redistribution (left–wing)*	− 0.047***	− 0.046***	− 0.021***
	(0.006)	(0.006)	(0.006)
*Income*		0.032***	0.024***
		(0.006)	(0.006)
Constant	7.020***	6.860***	6.104***
	(0.033)	(0.044)	(0.114)
Control for Socio-eco variables	No	No	Yes
Control for Country	No	No	Yes
Observations	64,848	64,848	64,848
R-squared	0.002	0.002	0.067

The dependent variable is individuals’ belief that hard work brings success*.* The question asked to respondents is: “In the long run, hard work usually brings a better life" vs. "Hard work doesn’t generally bring success—it’s more a matter of luck and connections.” This variable ranges from 1 to 10, 10 being that hard work brings success

For *Support for Redistribution,* the question is: “In political matters, people talk of left and right. How would you place your views on this scale, generally speaking?” This variable ranges from 1 to 10, 10 being the most leftist, interpreted as being the most in favor of redistribution

For *Income*, the question is: “On this card is an income scale on which 1 indicates the lowest income group and 10 the highest income group in your country. We would like to know in what group your household is”

The “Socio-eco” variables are gender, age, employment status, education, marital status and number of children

All variables are from the *World Value Survey* from 2005 to 2014. The sample comprises individuals from all democratic countries available in the dataset where the use of the political orientation as a proxy for the support for redistribution makes sense

OLS estimates are reported, with *t* statistics in parentheses (* significant at 10 percent; ** significant at 5 percent; *** significant at 1 percent)

A key difficulty in studying self-serving biases empirically is that we do not know the direction of the negative relationship between redistribution and beliefs about the source of income. In addition, observational data would not allow me to distinguish whether people engage in self-deception because of overconfidence and self-image considerations or to justify supporting personally advantageous redistributive policies. My approach thus consists of designing a laboratory experiment where individuals face different incentives to engage in motivated reasoning, and then measuring how this affects their perception of deservingness as explained in the next section.

## Experiment

I conduct a laboratory experiment in which high- and low-income individuals state their beliefs about the causes of their financial outcome. The experiment varies individuals’ incentives to engage in motivated reasoning to measure how this affects their perception of deservingness.

The experiment has three parts. In Part I, participants perform a work task and participate in a lottery, after which they receive either a high or low income. However, they do not know whether their income resulted from their work performance or luck in the lottery. In Part II, participants are asked to guess the causes of their income. They are incentivized to be accurate. In this part, the design varies whether or not subjects know that they will subsequently have to implement a redistributive policy. In Part III, they have the possibility to redistribute money from the participants with a high income to the participants with a low income.

### Experimental design

At the beginning of the experiment all participants are informed that the study has three parts. However, they discover the content of each part sequentially, i.e., they receive the instructions of each part at the beginning of each part.

*Part I* At the beginning of this part, all participants are randomly paired, and the pairing remains fixed during the entire study (i.e., for Parts I, II and III). Part I consists of two stages: a Work Task and a Lottery.

In the Work Task, participants have the opportunity to work on a simple task. Specifically, they are asked to count the number of times the number one (“1”) appears in a series of grids (similarly to Abeler et al., 2011). Their goal is to solve as many grids as possible in 20 min.^12^ The total number of grids solved represents a subject’s “Work Performance.” At the end of the Work Task, the computer compares the Work Performance of the paired participants. Whoever has the higher Work Performance in the pair is labeled the “winner” of the Work Task. Note that participants do not know their own absolute or relative performance levels and thus do not know whether they won the Work Task.

In the Lottery stage, a random draw determines, with equal probability, one of the two paired participants as the Lottery winner. As with the Work Task, participants do not know which participant won the Lottery.

At the end of Part I, the computer randomly selects one of the two stages, the Work Task or the Lottery, to determine the earnings in a pair. Once the computer determines the stage that counts, the winner of the selected stage receives income of CHF 30 while the paired participant receives CHF 0.

Importantly, this random draw is based on a compound lottery in which the computer first randomly draws a probability for each pair. This corresponds to the probability that Work Task, rather than the Lottery, determines a pair’s earnings. Specifically, at the beginning of Part I, the computer draws an integer from 0 to 100 with uniform probability for each pair. Participants are told that this is the “percent chance” that the Work Task, rather than the Lottery, determines the pair’s earnings. To give participants an intuitive representation of this probability, I also used the example of balls in an urn. Specifically, participants are asked to imagine that they have been assigned an urn containing 100 balls. Each ball may be either blue, in which case it corresponds to Work Performance, or it may be red, in which case it corresponds to luck in the Lottery. They are told that one ball is randomly drawn from their urn and this ball’s color determines whether the earnings in their pair are based on Work Performance (blue) or luck in the Lottery (red). Thus, the number of blue balls in their urn corresponds to the percent chance that the Work Task determines the earnings in their pair, rather than the Lottery.

At the end of Part I, Participants are only informed about their income and the income of their paired participant. However, they do not know the probability that was assigned to their pair, nor do they know whether the subsequent draw selected performance in the Work Task or luck in the Lottery to determine those earnings.

*Part II* This part elicits the beliefs that are central to this study. Specifically, participants are asked to guess how likely it is that the incomes in their pair were determined by the Work Task versus luck. That is, they have to guess the probability that the computer randomly assigned to their pair at the beginning of Part I. Participants provide an estimate between 0 and 100, corresponding to the percent chance that performance in the Work Task, rather than the Lottery, determined earnings in their pair. To incentivize truthful reporting of beliefs, I use the “matching probabilities” method.^13^ This method is a variation of the Becker et al. (1964) method adapted to elicit probabilities, rather than willingness to pay, in an incentive compatible way, regardless of risk preferences. By reporting their beliefs, participants indicate for which probability *p* they would be indifferent between receiving CHF 4 with the actual probability that performance in the Work Task determined earnings in their pair and receiving CHF 4 with probability $p$. For payment, the computer draws a random probability,$x$, from the uniform distribution. If $p > x$, she plays a lottery that pays out CHF 4 with the actual probability that the Work Task determined earnings in their pair. If $p \le x$, she plays a lottery that pays out CHF 4 with probability $x$. According to this method, participants have the highest chance to earn CHF 4 if they report the percent chance they believe is true (see Karni, 2009 for more details). The description of this method to participants is provided in dashed frame in Instructions for Part II in Appendix H in ESM.

**Fig. 1:**
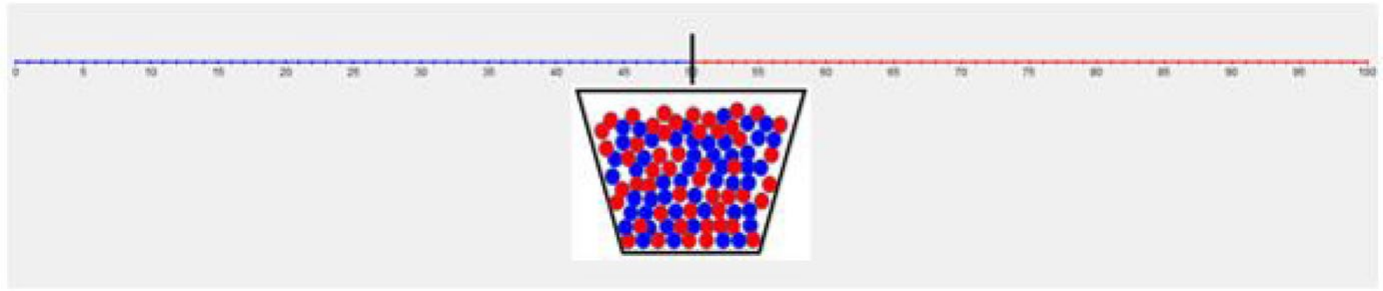
Screenshot of the slider. As an example, the position of the slider above indicates 50 percent chance that work determines income, corresponding to 50 blue balls in the urn

To report their guess, participants move a slider along a bar representing the percent chance, which ranges from 0 (very left end of the slider) to 100 (very right end of the slider). This design has the specificity to allow for the full spectrum of beliefs from entirely determined by luck to entirely determined by work. In other terms, asking participants to guess this probability, and not the task that has been selected, provides me with a finer scale of participants’ belief about the source of income. As they adjust the slider to the percent chance they believe is true, the number of blue balls in an urn of 100 balls on the screen changes to illustrate their choice of the percent chance. Figure 1 provides a screenshot of the slider and the urn.

*Part III* In this part, participants have the possibility to transfer income from the participant who received CHF 30 to the one who received CHF 0. Specifically, the participants who received CHF 30 in Part I decide on the transfer between 0 and 15 to implement within their pair. This transfer is then implemented. The participants who received CHF 0 in Part I cannot choose a transfer or influence the transfer chosen by their paired (high-income) participant. Instead, they are asked to predict how much they think the latter will redistribute (this question is not incentivized).

*Treatment variation* Remember that, at the beginning of the experiment, all the participants are only instructed about Part I, though they are informed that the study consists of three parts. My treatment variation consists of varying whether participants are informed that they will have the possibility to redistribute income in Part III before reporting their beliefs about the source of income in Part II.^14^ Note that transfers and beliefs are likely to be endogenous. Creating an exogeneous variation in expected allows me to study the causal impact of transfers on beliefs about the source of income.

In an *Information* condition, participants are given a summary of Part III at the end of Part I. Specifically, when informed about their income, they are also informed that in Part III, participants who received CHF 30 will have the opportunity to transfer some of their income to the participant who received CHF 0.^15^ They also receive a reminder of this possibility at the end of the instructions of Part II.^16^ This allows participants to engage in self-deception about the source of their income at the time of forming beliefs in Part II, in order to make it easier to subsequently propose transfers that are more personally beneficial. Specifically, being informed about the redistribution stage allow high-income participants to potentially exaggerate the extent to which they believe income is due to work in order to justify redistributing less to low-income participants.

In a *No Information* condition, participants are only instructed about Part III at the beginning of Part III. Therefore, they are not aware of the possibility to redistribute when stating their beliefs about the source of income in Part II. Thus, these beliefs cannot be formed self-servingly in order to justify implementing a favorable transfer.

Note that I implement this treatment variation—rather than, for example, varying participants’ ability to redistribute as Di Tella et al. (2015)—in order to be able to compare redistributive decisions in Part III across treatments.^17^

### Procedure

In total, 380 participants took part in this study. Half of them were allocated to the *Information* condition and the other half to the *No Information* condition. Subjects were students from the University of Zurich and the Swiss Federal Institute of Technology. I recruited them using the software h-Root (Bock et al., 2014) and conducted the experiments using the software z-Tree (Fischbacher, 2007) in October 2018.

At the beginning of each part, participants received written experimental instructions (provided in the Appendix H in ESM). In addition, an audio file with a summary of these instructions was played with intent to establish common information regarding the experiment. Comprehension questions had to be answered correctly before starting each part. At the end of the study, participants completed a questionnaire on their socio-economic background, their attitudes toward redistributive policies and their views regarding real-life determinants of economic success. The list of all elicited variables, their description and summary statistics are provided in Appendix B in ESM.^18^,^19^ The timeline of the experiment is presented in Figure E.1 in Appendix E in ESM.

I preregistered the data collection and analysis (AEARCTR-0003373). I initially aimed for a sample size of 192 participants but could recruit 190 participants. According to my simulations, the statistical power with this sample size was 80% to detect an effect size equal to 0.42 using a Wilcoxon-Mann–Whitney test (two tails) with normal parent distribution (Faul et al., 2007).^20^ I conducted 12 sessions (with about 32 participants per sessions), with each session lasting approximately 1.5 h. On average, participants earned CHF 38 (about 38 USD), which included a show up fee of CHF 10.

## A simple model

The standard economic assumption is that beliefs arise independently of the individuals’ self-interest. However, previous psychological and economic studies report that individuals manipulate beliefs in a self-serving way as reported in Sect. 2. To guide the analysis and the interpretation of the results, I use a simple model to provide a framework for developing hypothesis for the experiment.

### Timing and information structure

**Fig. 2:**
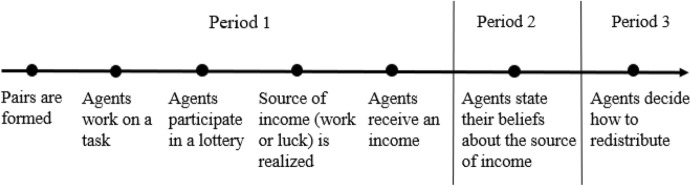
Timing

I first consider a model with N pairs of agents, where $i \in \left[ {1, \ldots , N} \right]$ denotes one pair. Each agent participates in three periods. The timing of events is shown in Fig. 2.

At the start of period 1, agents work on a task (work stage) that determines their work performance. They then participate in a lottery (lottery stage). One of these stages is randomly selected to determine the agents’ income in a pair. If the work (resp. lottery) stage is selected, the agent in the pair with the highest work performance (resp. best luck) receives a high income ($\overline{I}$) and the other agent receives a low income ($\underline {I}$). This means that, in each pair, one agent receives the high income, and the other agent receives the low income such that pair’s income, $I_{P}$, is equal to $\overline{I} + \underline {I}$. Agents are informed about the income they have received. However, none of them knows which stage has been selected, i.e., whether their income depends on their work performance or pure luck. What they know is the probability that work (W) rather than luck (L) determines income. This probability is denoted $p\left( W \right),$ with $p\left( W \right) \in \left[ {0, 1} \right]$. The probability that luck rather than work determines income is denoted $p\left( L \right)$, with *p* (L) = 1 − *p* (W). At the end of period 1, agents might receive some information about period 3. This information varies exogenously. In one case (*Information* condition), agents are informed that they will have the possibility to redistribute from high-income to low-income agents in period 3. In another case (*No Information* condition), they are not informed about this possibility.

In period 2, after they received their income, each agent states her belief about the probability that work rather than luck determined incomes. The probability reported by the high-income agents is denoted $\overline{p}_{i} \equiv p_{i} (W|\overline{I})$ and the probability reported by the low-income agents is denoted $\underline {p}_{i} \equiv p_{i} (W|\underline {I} )$, with $\overline{p}_{i} ,\underline {p}_{i} \in \left[ {0, 1} \right]$.

In period 3, each high-income agent decides how much to redistribute pair’s income $I_{P}$ in their pair. Specifically, she decides on an amount of income $I_{P} - t$ for herself and $t$ for the low-income agent. Following Cappelen et al. (2007), I assume that this decision depends on her concern for income and fairness. I denote $t_{i}^{F}$ the amount the high-income agent thinks is fair to redistribute to the low-income agent. A high-agent’s utility is given by the function $\overline{U}\left( {t_{i} } \right)$ such that:

1 $$\overline{U}\left( {t_{i} } \right) = { }\gamma_{i} \left( {I_{P} - t_{i} } \right) - {\upbeta }_{i} \frac{{\left( {t_{i} - t_{i}^{F} } \right)^{2} }}{{2I_{P} }}$$

where $\gamma_{i} > 0$ and ${\upbeta }_{i} \ge 0$ represent the weights the high-income agent attaches to income and to fairness consideration. Focusing the analysis on an interior solution, the optimal level of income $t_{i}^{*}$ to redistribute is given by:

$$t_{i}^{*} = t_{i}^{F} - \frac{{{ }\gamma_{i} }}{{{\upbeta }_{i} }}I_{P}$$

Consistently with previous studies discussed earlier, individuals’ perception of a “fair” allocation depends on their belief about the source of income. For the sake of simplicity, I assume that if an agent believes that work determines income, she thinks that it is fair not to redistribute. If an agent believes that luck determines income, she thinks it is fair to equalize income between a high-income and a low-income participant such that each of them receives $\frac{1}{2}I_{P}$.,^21^,^22^ The income the high-income agent thinks is fair to transfer thus depends on her belief about the probability that work rather than luck determined income such as $t_{i}^{F} = \left( {1 - \overline{p}_{i} } \right)\frac{1}{2}I_{P}$. The optimal level of transfer made by a high-income agent in pair $i, t_{i}^{*} ,$ is thus given by:

2 $$t_{i}^{*} = I_{P} \left[ {\frac{1}{2}\left( {1 - \overline{p}_{i} } \right) - \frac{{{ }\gamma_{i} }}{{{\upbeta }_{i} }}} \right]$$

The optimal level of transfer depends on each high-income agent’s beliefs about the source of income.

### No information about the possibility to redistribute

Consider the case in which agents are not informed about the possibility to redistribute in period 3, corresponding to the *No Information* condition in the experiment.

I first examine agents’ belief about the probability that income is due to work rather than luck in period 2. To determine such probability, agents update their prior beliefs $p\left( W \right)$ after observing their income $I$ using Bayes' rule. Agents’ posterior belief is thus given by:

$$p(W|I){ } = \frac{p\left( W \right) \times p(I|W)}{{p\left( W \right) \times p\left( {I|W} \right) + p\left( L \right) \times p(I|L)}}$$

where $p(I|L)$ represents their beliefs about the probability to receive a certain income when only luck matters and $p(I|W)$ when only work matters.

In this experiment, each stage is equally likely to be selected such that $p\left( {\text{W}} \right) = p\left( {\text{L}} \right) = 0.5$.^23^ In addition, all participants know that they have the same chance to receive a high-income in the lottery, such that $p(\overline{I}|L) = p(\underline {I} |L) = 0.5$. Their posterior belief thus only depends on their belief about the probability they receive a high income in the work stage $p_{i} \left( {\overline{I}|W} \right)$. Under *No Information*, the probability stated by high-income agents is given by:

$$\overline{p}_{i}^{NI} \equiv p_{i} (W|\overline{I}) = \frac{{0.5 \times p_{i} (\overline{I}|W)}}{{0.5 \times p_{i} (\overline{I}|W) + 0.25}}$$

and the probability reported by low-income agents is:

$$\underline {p}_{i}^{NI} \equiv p_{i} (W|\underline {I} ) = \frac{{0.5 \times (1 - p_{i} (\overline{I}|W))}}{{0.5 \times (1 - p_{i} (\overline{I}|W)) + 0.25}}$$

Let $p\left( {\overline{I}|W} \right)$ be a continuous random variable with range [0,1] and probability density function $f_{P} \left( p \right)$. When agents are rational, i.e., do not form biased beliefs, I assume that their belief about their ability to win the work task follows a symmetric (e.g., Gaussian or bimodal) distribution around a mean of 0.5. In other terms, $p\left( {\overline{I}|W} \right)$ is distributed according to a symmetric distribution with mean 0.5. The expected value of $p\left( {\overline{I}|W} \right)$ is thus equal to 0.5. Indeed, most individuals cannot rationally evaluate themselves as better (or worse) than average, i.e., think that they have either a higher (or lower) probability to be the winner in the work task in the context of this experiment. Since the probability density function (PDF) is symmetric around 0.5, we have $f_{P} \left( p \right) = f_{P} \left( {1 - p} \right)$. As a result, high-income and low-income agents’ expected value should be equal when they evaluate themselves without bias such that $E\left[ {\overline{p}_{i}^{NI} } \right] = E\left[ {p_{i}^{NI} } \right]$ (see Proof B in Appendix F in ESM).

However, we know from previous literature that individuals have biased beliefs about their ability, i.e., they think that they are better than average such that $E\left[ {p_{i} \left( {\overline{I}|W} \right)} \right] > 0.5$. This is usually interpreted as overconfidence or a desire for a positive self-image.^24^ When $E\left[ {p_{i} \left( {\overline{I}|W} \right)} \right] > 0.5$, the probability that the work stage determined income reported by high-income agents is higher than the probability reported by low-income agents such that $E\left[ {\overline{p}_{i}^{NI} } \right] > E\left[ { \underline {p}_{i}^{NI} } \right]$. Intuitively, if an agent thinks she is more likely to receive a high income in the work task and if she actually receives a high income, she will then believe that the probability that her income is due to work is higher than if she receives a low income (in this later case, she thinks that it is more likely to be due to bad luck).

#### Hypothesis H1

Individuals believe to a larger extent that their income is due to work, rather than luck, when they receive a high income than when they receive a low income.

I now examine how much high-income agents redistribute in period 3. Under *No Information*, the transfer made by a high-income agent to a low-income one can be written as $t\left( {\overline{p}_{i}^{NI} } \right) = I_{P} \left[ {\frac{1}{2}\left( {1 - \overline{p}_{i}^{NI} } \right) - \frac{{{ }\gamma_{i} }}{{{\upbeta }_{i} }}} \right]$ using (2). It follows that $dt\left( {\overline{p}_{i}^{NI} } \right)/d\overline{p}_{i}^{NI} < 0$, i.e., the higher the belief about the probability that work, rather than luck, determined income $\overline{p}_{i}^{NI} ,$ the lower the transfer $t\left( {\overline{p}_{i}^{NI} } \right).$

#### Hypothesis H2

The more high-income individuals believe that income is due to work rather than luck, the less they redistribute.

### Information about the possibility to redistribute

I now consider the case in which agents are informed about the possibility to redistribute in period 3, corresponding to the *Information* condition.

I first examine agents’ belief about the probability that income is due to work rather than luck in period 2. Agents can form self-serving beliefs about the source of income to implement the transfer that is the most advantageous to themselves. A high-income agent’s utility under *Information*, is given by the function $U\left( {\overline{p}_{i} } \right)$ such that:

$$U^{I} \left( {\overline{p}_{i} } \right) = \gamma_{i} \left[ {1 - \frac{1}{2}{ }\left( {1 - \overline{p}_{i} } \right) + \frac{{{ }\gamma_{i} }}{{{\upbeta }_{i} }}} \right]I_{P} - \frac{{{ }\gamma_{i}^{2} }}{{2{\upbeta }_{i} }}I_{P} - \mu \frac{{(\overline{p}_{i}^{NI} - \overline{p}_{i} )^{2} }}{2}$$

The first two terms represent agent’s earnings after Period 3, and it is obtained by substituting (2) into (1). To maximize her earnings, an agent might want to convince herself that income is certainly due to work ($\overline{p}_{i} = 1$). However, engaging in a self-serving evaluation of $\overline{p}_{i}$ is costly, similarly to Rabin (1994), Konow (2000) and Di Tella et al. (2015). The cost of dissonance is represented by the last term of the above utility function.^25^ The higher they distort their Bayesian updated beliefs $\overline{p}_{i}^{NI}$ in the direction of beliefs they are motivated to hold, the higher the cost of dissonance. The parameter $\mu$ represents the extent to which agents care about holding dissonant beliefs, with $\mu > 0$.

I focus my analysis on interior solutions and on cases in which $\mu < \infty$, where agents engage in self-deception.^26^ Hence, the beliefs they hold under *Information*, $\overline{p}_{i}^{I}$, are given by the first-order condition such that:$\overline{p}_{i}^{I} = \overline{p}_{i}^{NI} + \frac{{\gamma_{i} }}{2\mu }I_{P}$. It follows that $\overline{p}_{i}^{I} > \overline{p}_{i}^{NI}$ indicating that high-income agents exaggerate the extent to which income is due to work rather than luck in *Information* with respect to *No Information*.

#### Hypothesis H3a

The extent to which high-income individuals believe that income is due to work, rather than luck, is greater when they know that redistribution will subsequently occur.

I now examine how much high-income agents redistribute income in period 3. If high-income participants report a higher probability that the Work Task, rather than the Lottery, determined income in *Information* than in *No Information*, they will redistribute less in *Information* than in *No Information,* since $t\left( {\overline{p}_{i}^{I} } \right) < t\left( {\overline{p}_{i}^{NI} } \right).$

#### Hypothesis H3b

High-income individuals redistribute less when they adopt self-serving beliefs about the probability that income is due to work, rather than luck.

## Results

The main focus of this paper is whether people form their beliefs about the source of income inequality in a self-serving manner, and how these beliefs influence redistribution. I first compare beliefs about the probability that the Work Task determined income in a pair among high-income and low-income participants in the treatment *No Information*. I also examine whether these beliefs influence how much participants redistribute. Turning to the principal focus of the study, I then compare beliefs regarding the roles of work versus luck held by the high-income participants between the *No Information* and *Information* conditions, and the subsequent transfers they implement.

### High-income versus low-income participants in *no information*

**Fig. 3:**
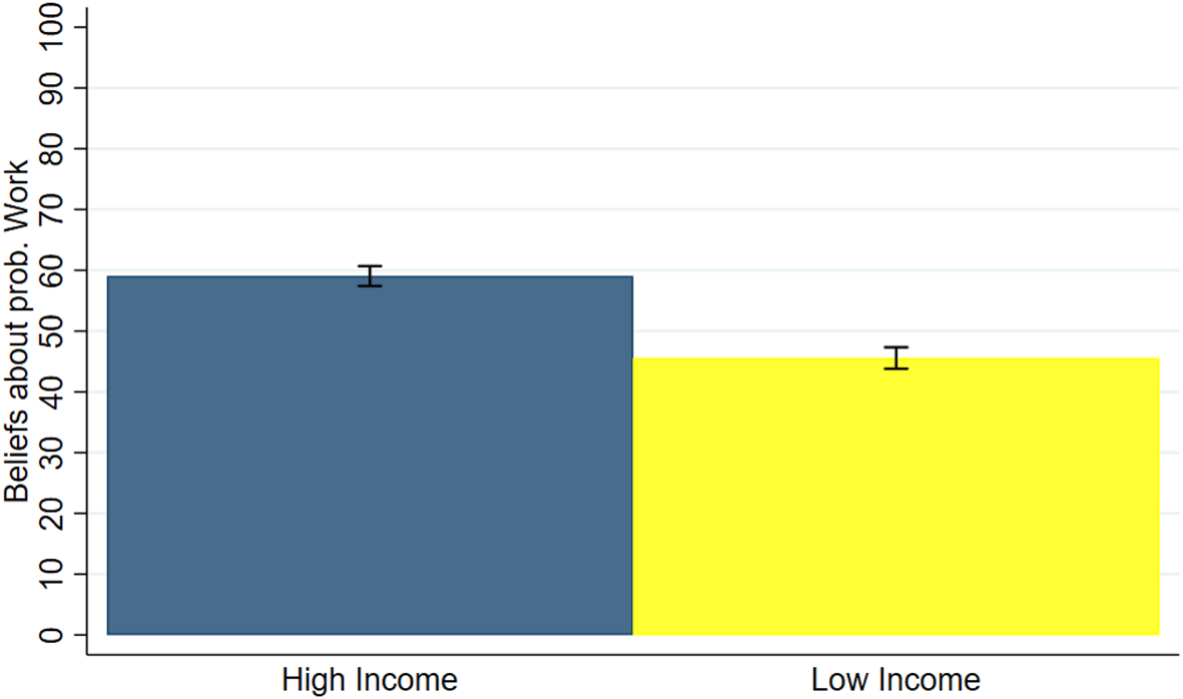
Average beliefs by income. “Beliefs about Prob. Work” on the y-axis represents the elicited beliefs about the probability that the Work Task determined income rather than the Lottery. Data concerns the treatment *No Information.* Error bars denote the standard error of the mean

In this sub-section, I focus on results in the *No Information* condition—i.e., when participants do not have any monetary motive to engage in self-deception. I first compare beliefs regarding the roles of work performance versus luck among the participants who received CHF 30 and those who received CHF 0. Figure 3 presents the belief that Work determined income reported by high-income and low-income participants. This reveals that beliefs differ systematically between those who obtained a high income and those who did not. On average, high-income participants believe that there is a higher chance that their Work Performance determined earnings in their pair (about 59 percent) than low-income participants (about 46 percent). This difference is statistically significant using the non-parametric Wilcoxon rank-sum test at the individual level (*p* < 0.0001). Hypothesis H1 is supported by the data.

On average, participants attribute the income in their pair to a larger extent to work when they received a high-income, and to luck when they received a low income. Using a two-tailed t-test, I find that the mean beliefs reported by high-income and low-income participants are statistically different from 50 (*p* < 0.0001 for high-income participants and *p* = 0.0138 for low-income ones). This result confirms previous findings that people tend to attribute their success to factors within their individual control, and their failure to factors beyond individual control (e.g., Miller & Ross, 1975; Weiner, 1985).

Consistently with my model, this result suggests that participants think that they are more likely to win the Work Task. This is supported by the answers to the post-question about their probability that their Work Performance is in the top half of performances in their session (see Question 1 in Appendix D in ESM). Both high- and low-income participants believe that they have a higher chance to win the Work Task irrespectively on whether they received a high or low income. This is consistent with the idea that people are overconfident, i.e., they think they are better than average.

I now examine whether these beliefs influence how much high-income participants transfer to low-income ones. The simple correlation coefficient between the transfers implemented and beliefs regarding the roles of work performance versus luck held by those who received CHF 30 is − 0.21 and is statistically significant (*p* = 0.043).^27^ This reveals a negative relationship: the more participants believe that Work Performance is the source of income inequality, the less they redistribute. Hypothesis 2 is supported by the data.

The results of this sub-section confirm the previous well-established findings that people take more responsibility for economic success than failure, and that their perception of how much a success or failure is deserved influence their decision whether to support redistribution.

### High-income beliefs in *no information* versus *information*

I now compare beliefs about the probability that the Work Task, rather than the Lottery, determined income in a pair in *No Information* and *Information*.

**Fig. 4:**
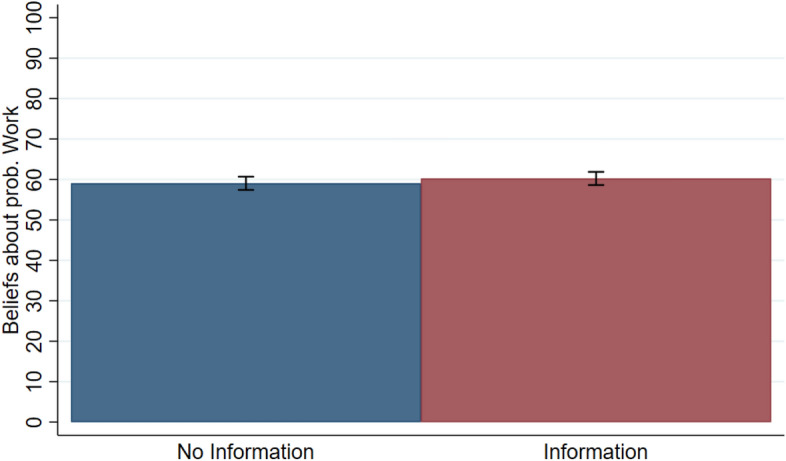
Average high-income beliefs across treatments. “Beliefs about Prob. Work” on the y-axis represent the elicited beliefs about the probability that the Work Task determined income rather than the Lottery. Error bars denote the standard error of the mean

Figure 4 shows the average beliefs of high-income participants in both treatments (about 59 percent in *No Information* and 60 percent in *Information*). This figure reveals that beliefs increase only very slightly in *Information* and this difference is statistically insignificant using a two-sided Wilcoxon rank-sum test (*p* = 0.73). High-income participants do not overestimate the extent to which income is due to work when they know that they may be financially harmed by redistribution at the time they form their beliefs. Thus, Hypothesis *H3a* is not supported by the data.

Table 2 presents results from OLS regressions that complement these observations. The dependent variable is the beliefs about the probability that the Work Task, rather than the Lottery, determined income in a pair stated by high-income participants. In model (1), I include the binary treatment variable, *Information*, taking on value 1 if this treatment is implemented and 0 if *No Information* is implemented. Model (2) repeats this analysis but controls for gender, age and field of study, and for session fixed effects. In both models, the positive coefficient for *Information* indicates slightly increased beliefs, but the impact is small and not statistically significant. This confirms that high-income participants do not engage in self-deception concerning the determinant of their income when they are aware of the redistributive possibility. Moreover, the very small size of the coefficients indicates that the effects are not only statistically insignificant, but also negligible in magnitude.

**Table 2:** OLS regressions of high-income beliefs

	(1)	(2)
*Information*	1.200	1.761
	(2.313)	(2.602)
Constant	59.053***	54.034***
	(1.635)	(10.887)
Control for gender, age & field	No	Yes
Control for sessions	No	Yes
Observations	190	190
R-squared	0.001	0.110

OLS estimates are reported, with standard errors in parentheses (* significant at 10 percent; ** significant at 5 percent; *** significant at 1 percent)

**Fig. 5:**
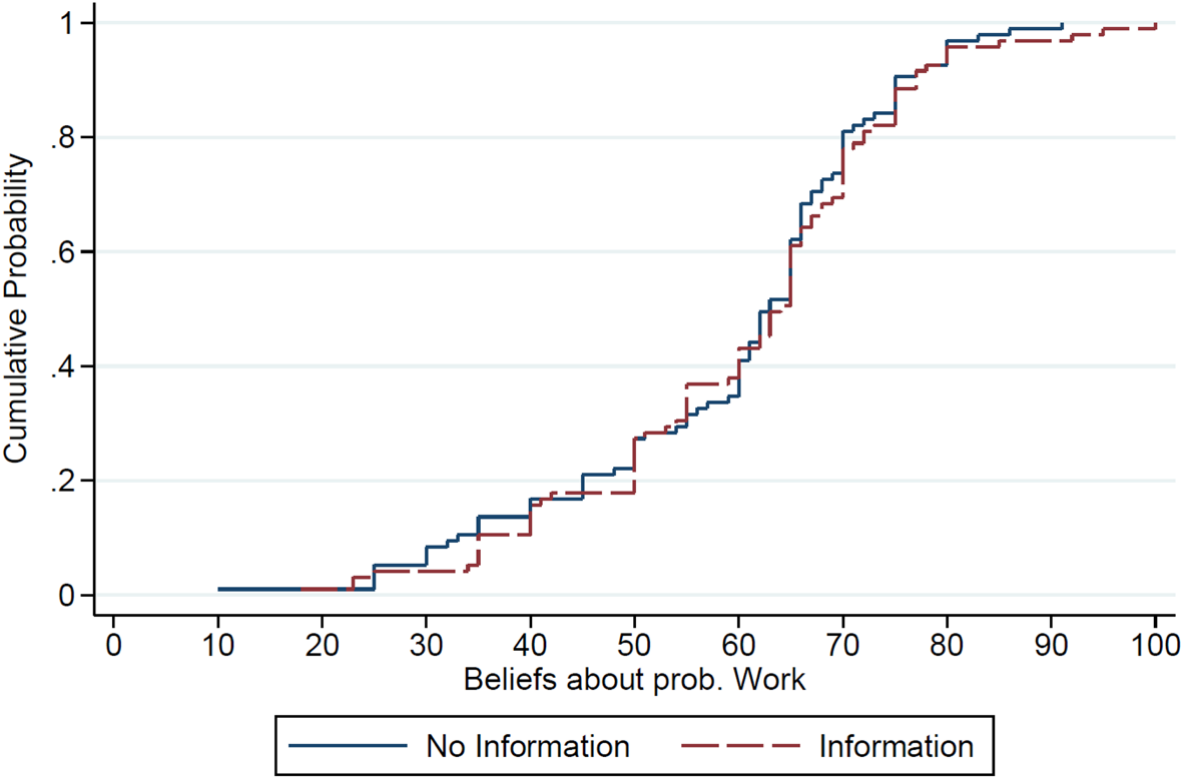
Cumulative distribution of high-income beliefs

Furthermore, Fig. 5 shows the empirical cumulative distributions (CDFs) of high-income beliefs in treatments *No Information* and *Information*. This reveals that both distributions are fairly similar, as confirmed by the two-sample Kolmogorov–Smirnov test which cannot reject the equality of distributions between both treatments (*p* = 0.991). Moreover, the CDFs reveal that less than 28 percent of high-income participants think that there is at least 50 percent chance that their income was due to luck. This suggests that about 72 percent think they have a higher chance to be better than their paired participants in the Work Task.

### Redistribution in *no information* versus *information*

**Fig. 6:**
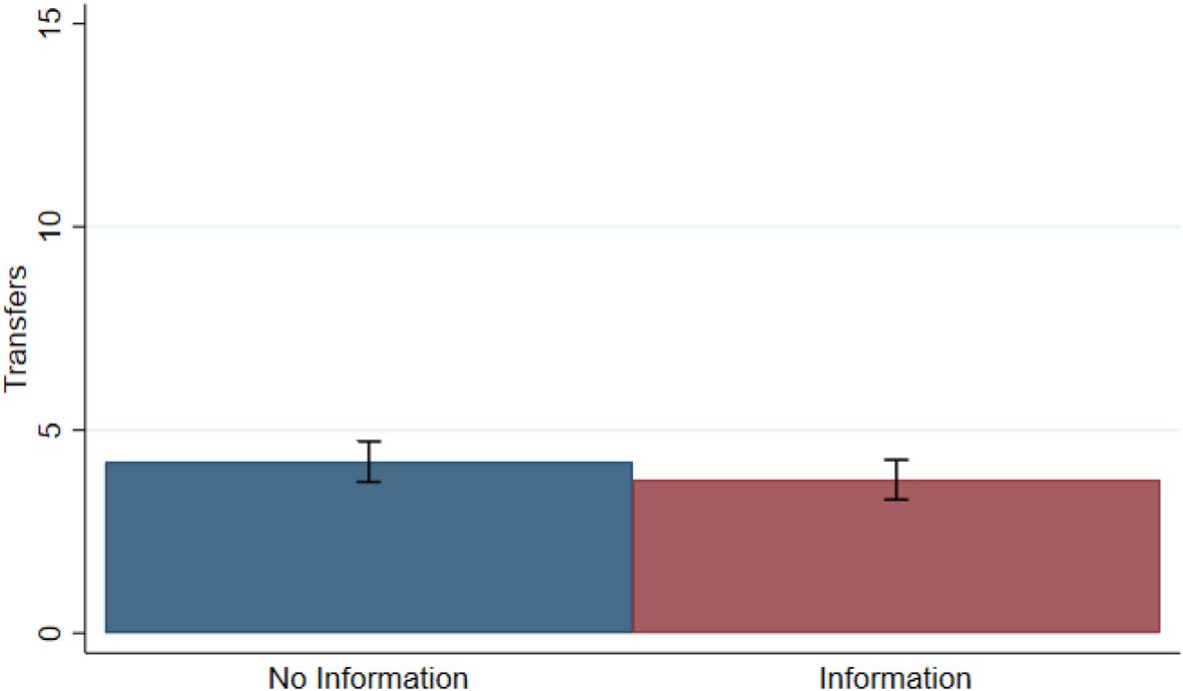
Average transfers across treatments. “Transfers” on the y-axis represent the transfers implemented by the high-income participants. Error bars denote the standard error of the mean

Next, I compare how much high-income participants transferred to low-income ones in *No Information* and *Information*. Figure 6 presents the average transfers in both treatments. This indicates that transfers decrease only very slightly in *Information*. However, this difference is not statistically significant using a two-sided Wilcoxon rank-sum test (*p* = 0.483).^28^ Hypothesis *H3b* is thus rejected by the data. This is consistent with the previous result: if high-income participants hold the same beliefs in both treatments, they should redistribute to the same extent in *No Information* and *Information*.

Regressions of the transfers implemented in Table 3 confirm this result. In model (1), I include the binary treatment variable *Information*. Model (2) repeats this analysis but controls for gender, age and field of study, and for session fixed effects. Model (3) introduces the variable *Beliefs about probability of Work* which represents beliefs about the probability that the Work Task, rather than the Lottery, determined income in a pair stated by high-income participants. In all models, the negative coefficient for *Information* indicates that transfers slightly decrease under the *Information* condition, but the magnitude is small and not statistically significant. This confirms that high-income participants do not transfer significantly less money when they know about the possibility to redistribute in advance.^29^ Importantly, in model (3), the coefficient on *Beliefs about probability of Work* is negative and statistically significant. This reveals that the more high-income participants believe that the income in their pair is more likely due to work rather than luck, the less they subsequently transfer money to the low-income participants. This is consistent with previous findings that beliefs about the source of income influence the willingness to redistribute.

**Table 3:** OLS regressions of transfers

	(1)	(2)	(3)
*Information*	− 0.442	− 0.407	− 0.304
	(0.700)	(0.785)	(0.774)
*Beliefs about probability of work*			− 0.058***
			(0.023)
Constant	4.221***	7.506**	10.637***
	(0.495)	(3.285)	(3.460)
Control for gender, age & field	No	Yes	Yes
Control for sessions	No	Yes	Yes
Observations	190	190	190
R-squared	0.002	0.116	0.148

OLS estimates are reported, with standard errors in parentheses (*significant at 10 percent; **significant at 5 percent; ***significant at 1 percent)

## Conclusion

Redistribution is a topic of central importance in economics. Prior research recognizes that individuals’ perceptions of the deservingness of those who are impacted by redistribution can play a critical role in determining support for different redistributive policies. However, little is known about how perceptions of deservingness are formed. This paper investigates a largely unexplored aspect of how individuals’ support for redistribution is formed—in particular, whether people evaluate the causes of income inequality in a manner that justifies supporting the most financially favorable redistributive policies. Despite the intuitive appeal that it might be the case, which would also be consistent with the literature on motivated reasoning, I do not find that people identify the causes underlying economic inequality self-servingly for financial gain. People distort their perception of deservingness because they are overconfident, but the financial incentive of benefitting of an advantageous redistributive system does not make this distortion any stronger. This is important to know because people might believe that redistribution harms them unfairly—a belief they are particularly susceptible to hold if they think people have biased beliefs regarding deservingness—then such grievances may be manifested in their interactions with others in society, particularly with those who are believed to have benefitted unfairly from redistribution. This can have significant consequences for the degree of social conflict in a society.

In addition, this study also serves as an important example of where people with a financial incentive to engage in belief manipulation do not do so. This is important to document as we might believe from previous literature on motivated beliefs (see, for instance, Gino et al., 2016 for a review) that people *systematically* engage in self-deception for financial gain.

As a possible avenue for follow-up research, it would be interesting to examine situations where low-income participants decide on redistribution as well. This could help to understand the voting behavior of low-income voters (that typically constitute a substantial share of the electorate) over income redistribution. A priori, whether the (null) effect I identified for high-income individuals would be different for low-income individuals is not obvious. I leave the analysis of the redistributive decisions of low-income individuals for future research. It would also be interesting to examine situations where people do not have any information at all about the source of income. We know from Haisley and Weber (2010) that situations with more ambiguity allow a greater degree of self-serving bias. This suggests that we could observe some motivated beliefs about the source of income for redistributive purposes in this setting.

## Supplementary Information

Below is the link to the electronic supplementary material.

Supplementary file1 (DOCX 498 kb)

## Footnotes

1 A similar idea has been articulated by Krugman (2014): “Many influential people have a hard time thinking straight about inequality. Partly, of course, this is because of Upton Sinclair’s dictum: it’s hard for a man to understand something when his salary depends on his not understanding it. Part of it is because even acknowledging that inequality is a real problem implicitly opens the door to taking progressive policies seriously.”

2 Cappelen et al. (2019) also consider situations in which the source of inequality is uncertain such that unequal incomes can either reflect differences in performance or in luck. In their paper, they examine impartial spectators’ redistributive decisions—and thus formed without self-interest—in such context.

3 Varying the time at which participants learn about their own incentives to study self-serving bias has been used previously for instance in Babcock et al. (1995) and Gneezy et al. (2020).

4 Furthermore, higher earners’ willingness to reduce inequality is important to understand as they can decide to redistribute not only though tax policies but also through charitable donation. It also has been argued that individuals with higher incomes are more likely to have a great political influence on tax policies as they are usually seen as more politically active and have a better access to legislators through lobbying or the financing political campaigns (Gilens, 2012).

5 If potentially surprising, this result is nevertheless in line with recent laboratory findings that show limits about where and when people engage in self-serving deception for financial gain (e.g., Van der Weele et al., 2014; Bartling and Özdemir, 2017 and Ging-Jehli et al., 2020). Moreover, this result is consistent with field data showing that Mormons do not bias their definition of what constitutes income for the purpose of tithing in the direction of their own financial self-interest (Dahl and Ransom, 1999).

6 For instance, Fong (2001) find that compared to the full sample, the high-income, upwardly mobile sub-sample has stronger average beliefs in self-determination.

7 One exception is Rey-Biel et al. (2018) that find that the beliefs about other’s source of income do not influence participants’ willingness to give. However, they suggest that it might be due to the fact that participants care more about the source of their own income in their decision whether to give.

8 These beliefs also play an important role in theoretical models examining the demand for redistribution (e.g., Piketty, 1995; Alesina and Angeletos, 2005; Bénabou and Tirole, 2006).

9 Deffains et al. (2016) also find that unsuccessful participants tend to attribute their failure to external factors and support more redistribution. However, the extent to which this is a self-serving bias is difficult to determine since these participants are actually right believing that it was beyond their individual control (they were assigned to the difficult task which did not offer any chance to outperform participants assigned to the easy task and thus to be successful).

10 Controlling for income is to make sure that the relationship between redistribution and beliefs about the source of income is not driven by the relationship between income and beliefs about the source of income. Indeed, we know from previous literature that (i) the less people support redistribution, the richer they are and (ii) the richer people are, the more they attribute success to effort because they are overconfident.

11 Comparing the estimates for the support for redistribution with the estimates for income allows for a better understanding of the economic relevance of people’s support redistribution as an explanatory variable. Indeed, we already know that income is an important driver of the beliefs about the source of income inequality (Deffains et al., 2016). In Model 2 in Table 1, the coefficient for income is 0.032 (*p* < 0.01) and -0.046 (*p* < 0.01) for support for redistribution. The effect of support for redistribution is thus 1.4 times larger than the effect of income. This suggests that the support for redistribution is at least as important as income.

12 Participants are told that, during the Work Task, they may take a break from solving grids, and stop working. However, they are asked to keep quiet while they take a break. This gives participants leeway in believing that other might be lazy and thus responsible for their failure if it is convenient for them to think so.

13 The “matching probabilities” method is also known as “lottery method”, “crossover mechanism,” and “reservation probabilities.” This method has been used by several recent studies including Möbius et al. (2017), Schlag et al. (2015) and Coutts (2019).

14 The idea that forcing people to develop judgments before they know their self-interest in doing so prevent them to distort their judgment in their favor is illustrated in experiments on pre-trial bargaining (Babcock et al., 1995; Babcock and Loewenstein, 1997).

15 The summary of instructions for Part III given at the end of Part I in *Information* is provided in Appendix A in ESM.

16 The instructions for Part II for both treatments are similar but for one sentence that reminds participants that Part III concerns redistribution in *Information*. The instructions for Part II can be found in Appendix H in ESM.

17 Di Tella et al. (2015) manipulate the incentives to engage in self-deception by varying the maximum of money that can be allocated between parties. When the maximum money that can be transferred is low (resp. high), the benefits from self-deception are low (resp. high) as well. This design implies that they cannot compare the money allocated in both treatments since the range of possible allocation is not the same (different maximum of money allocated).

18 In Appendix C in ESM, I examine the relationship between participants’ views regarding real-life determinants of economic success and their attitudes toward redistribution using the answers to the questionnaire. The results in Table C.1 show a significant and robust negative relationship between both variables which is consistent with the findings using observational data presented in Sect. 3.

19 After Part III, but before the questionnaire, participants were asked to respond to five questions about the experiment. Their description can be found in Table D.1 in Appendix D in ESM.

20 Note that two types of high-income participants have no incentive to distort their beliefs because their transfer do not depend on them, thus reducing my sample size of participants susceptible to engage in self-deception. First, one could think of those who would always keep all the money for themselves. In a meta-analysis, Engel (2011) estimates that about 36% of participants do not transfer anything in Dictator Games. If I exclude from my sample these participants, I am left with about 120 participants out of 190. In this case, the sample size allows me to detect an effect size of at least 0.53. Second, one could think of participants who have a fairness view that does not distinguish between the different sources of inequality contrarily to meritocrats (e.g., egalitarians or libertarians). Cappelen et al. (2019) find that over 75 percent of the participants in their laboratory experiment can be described as meritocrats. This suggests that for 90 out of 120 participants, the source of income inequality matters when deciding how to redistribute. Assuming a sample size of 90 participants, I can rule out the possibility that there is an effect size of at least 0.61. For comparison, a recent prominent study on motivated beliefs in the context of redistribution, Di Tella et al. (2015), estimates an effect size of 0.67 when beliefs are incentivized.

21 In my framework, I cannot apply the accountability principle following the idea of proportionality so that agents would receive income depending on their work (see for example Konow, 1996, 2000; Cappelen et al., 2007, 2010) since work performance is not observable. However, one could consider different versions of the accountability principle. For example, one could assume that high-income agents consider it fair to give something to low-income agents when income is due to work. The hypotheses derived from my model still hold in this case under the reasonable assumption that high-income individuals think it is fair to keep a higher share of total income when income is due to work (see Proof A in Appendix F in ESM).

22 Note that some people might be libertarian such that they do not support redistribution no matter the source of income, or egalitarian, i.e., they support an equal split of income no matter the source of income. However, a recent study by Cappelen et al. (2019) show that, for a vast majority of people, the source of income inequality matters for redistribution.

23 Participants are told in this experiment that $p(W)$ can take any value (integer) between 0 and 100 percent, with each value being equally likely to be drawn. The probability $p(W)$ is thus equal to 50 percent because of the Reduction of Compound Lottery axiom (RCLA) stating that compound lotteries and probabilistic equivalent simple lotteries are equivalent.

24 Benoît and Dubra (2011) argues that better-than-average behavior alone should not be interpreted as overconfidence. For example, drivers who have not had an accident should rationally evaluate themselves as better than average (because the probability to have a car accident is low over the entire population). However, because there is a priori no reason that participants in my experiment receive more positive signals about their abilities to win a work task than a negative one, I will interpret such better-than-average behavior as overconfidence in the rest of the paper.

25 This dissonance cost can be seen as a psychological cost and/or the cost of not receiving rewards from accurate beliefs in the elicitation task.

26 If μ → ∞, then it would never be optimal to engage in self-deception.

27 Figure G.1 in Appendix G in ESM illustrates the relationship between the transfers implemented and beliefs regarding the roles of work performance versus luck held by those who received CHF 30 in *No Information*.

28 This result is consistent with what low-income participants predicted when asked how much they think high-income participants will transfer—remember that, while high-income participants were deciding about the transfer they would like to implement, low-income participants were asked to predict how much they think will be redistributed. Their beliefs about redistribution in *No Information* and *Information* are not statistically significantly different (see Figure G.2 in Appendix G in ESM).

29 Table G.1 in Appendix G in ESM reports the results of a double-hurdle regression of the decision of whether to transfer and, conditionally, how much they transfer (such model has been used for example by Brañas-Garza et al., 2017; Engel, 2011; Erkal et al., 2011). Consistently with the results from the OLS regressions in Table 3, participants are not significantly less likely to transfer any money in *Information*. Furthermore, the conditional amount transferred is not significantly lower in *Information*. This confirms that high-income participants do not decide to redistribute differently when they know about the possibility to redistribute in advance.
