# Supplementary material for: Redistribution and beliefs about the source of income inequality
Source: Exp Econ. 2021 Sep 27;25(3):876–901. doi: 10.1007/s10683-021-09733-8 (PMC9165271; doi:10.1007/s10683-021-09733-8)
Supplement: Supplementary file 1 — Supplementary file1 (DOCX 498 kb) [file 10683_2021_9733_MOESM1_ESM.docx]

**Appendix**

1. **Summary of Instructions for Part III**

In the *Information* condition, participants are given the following summary of Part III at the end of Part I.

*Note that in Part III, the participant in the pair who received CHF 30 will have the opportunity to transfer some of these earnings to the paired participant who received CHF 0. More specifically, the participant who received CHF 30 will choose an amount to transfer between CHF 0 and CHF 15. This amount will then be transferred to the participant in the pair who received CHF 0. The participant who received CHF 0 will not be able to choose a transfer or to influence the transfer chosen by the participant who received CHF 30. This means that participants' final earnings will depend on their earnings in Part I and on the amount transferred in Part III by the participant who received CHF 30.*

*For the participant who received CHF 30 in Part I: Final earnings = CHF 30 - amount transferred;*

*For the participant who received CHF 0 in Part I: Final earnings = CHF 0 + amount transferred.*

1. **Questionnaire Data**

| **Table B.1**: Detailed description of the socio-economic variables | |
| --- | --- |
| Variable | Description |
| Female | 0- No (Male); 1- Yes |
| Age | In years |
| Study | Field of study |
| Nationality | Country of Nationality |
| Disposable income | *How much money (CHF) do you have at your disposal each month (approximately, after housing costs)?* |
| Relative income | *How do you think your income and financial situation currently compare to those of others in Switzerland who are of similar age?*  0 - *don't know / no answer,* 1 - *much below average,* 2 - *somewhat below average,* 3 - *about the average,* 4 - *somewhat above average,* 5 - *much above average* |
| Working hours | *How many hours do you work per week, alongside your studies (during the semester)?* |
| Parents’ education | *What is the highest degree or level of education completed by either of your parents?*  0 - *don't know / no answer,* 1 - *did not complete Medium school,* 2 - *Medium school,* 3 - *some college (i.e. university),* 4 - *bachelor's degree,* 5 - *master's degree,* 6 - *advanced graduate work or Ph.D.* |
| Family income | *Approximately, what was the highest total gross income obtained by your parents in any past year?*  0 - *don't know / no answer,* 1 - *under CHF 50'000,* 2 - *CHF 50'000 to 100'000,* 3 - *CHF 100'000 to CHF 150'000,* 4 - *CHF 150'000 to CHF 200'000,* 5 - *above CHF 200'000* |
| Social class | *To which social class do you think your parents belong?*  0 - *don't know / no answer*, 1 - *upper class*, 2 - *upper middle class*, 3 - *lower middle class*, 4 - *working class, 5 - lower class* |
| Future class | *Just your best guess, to which social class do you think you will belong in the future, say about ten years from now?*  0 - *don't know / no answer*, 1 - *upper class*, 2 - *upper middle class*, 3 - *lower middle class*, 4 - *working class, 5 - lower class* |
| Political orientation | *Where would you classify yourself on the left/right political spectrum?*  from 1 (left-wing) to 9 (right-wing). |
|  | |

| **Table B.2**: Description of the views about real-life determinants of economic success | |
| --- | --- |
| Variable | Description |
| View 1 | *Hard work does not generally bring success, it is more a matter of luck and connections.* (-) |
| View 2 | *The main cause of poverty is poor's lack of effort rather than bad luck.* |
| View 3 | *The main cause of wealth is rich's hard work rather than luck.* |
| View 4 | *There is plenty of opportunity in Switzerland today and anyone who works hard can go as far as they want.* |
| Note: For these questions, they have to select one of the seven answers which best describes their present agreement or disagreement with the statement, from "Completely disagree" to "Completely agree" (from -3 to 3). Note that *View 1* is reverse coded. | |

| **Table B.3**: Description of the redistributive attitudes | |
| --- | --- |
| Variable | Description |
| Attitude 1 | *Governments should redistribute wealth by taxing the rich.* |
| Attitude 2 | *Poor people should take more responsibility to provide for themselves.* (-) |
| Attitude 3 | *The fact that some people are rich and others are poor represents a problem that needs to be fixed through redistribution.* |
| Attitude 4 | *The distribution of money and wealth in Switzerland today is fair, and should not be more evenly distributed among a larger percentage of the people.* (-) |
| Note: For these questions, they have to select one of the seven answers which best describes their present agreement or disagreement with the statement, from "Completely disagree" to "Completely agree" (from -3 to 3). *Attitude 2* and *4* are reverse coded. | |

| **Table B.4**: Descriptive statistics | | | | | | |
| --- | --- | --- | --- | --- | --- | --- |
| Variable | N | Mean | SD | Min | Max |  |
| Female | 380 | 0.52 | 0.50 | 0 | 1 |  |
| Age | 380 | 23.68 | 3.26 | 18 | 42 |  |
| Disposable income | 371 | 1020.08 | 5225.34 | 20 | 99999 |  |
| Relative income | 371 | 2.14 | 1.00 | 1 | 5 |  |
| Working hours | 371 | 7.46 | 9.75 | 0 | 45 |  |
| Parents’ education | 370 | 3.82 | 1.55 | 1 | 6 |  |
| Family income | 303 | 2.57 | 1.54 | 1 | 5 |  |
| Social class | 363 | 3.24 | 0.93 | 1 | 5 |  |
| Future class | 365 | 3.78 | 0.68 | 1 | 5 |  |
| Political orientation | 380 | 3.98 | 1.75 | 1 | 9 |  |
| View 1 | 380 | 0.03 | 1.65 | -3 | 3 |  |
| View 2 | 380 | -0.89 | 1.56 | -3 | 3 |  |
| View 3 | 380 | -0.42 | 1.53 | -3 | 3 |  |
| View 4 | 380 | 0.37 | 1.64 | -3 | 3 |  |
| Real-life beliefs ^*^ | 380 | -0.00 | 0.80 | - | - |  |
| Attitude 1 | 380 | 0.94 | 1.61 | -3 | 3 |  |
| Attitude 2 | 380 | 0.24 | 1.53 | -3 | 3 |  |
| Attitude 3 | 380 | 0.29 | 1.61 | -3 | 3 |  |
| Attitude 4 | 380 | 0.37 | 1.55 | -3 | 3 |  |
| Redistributive attitude ^**^ | 380 | -0.00 | 0.83 | - | - |  |
| ^*^ I use a factor analysis on answers to questions about real-life determinants of economic success and create a univariate measure of the extent to which participants believe that economic success is due to hard work rather than luck, called *Real-life beliefs.*  ^**^ I use a factor analysis on answers to questions about redistribution and create a univariate measure of the extent to which participants support redistribution called *Redistributive attitude.*  *Notes.* For each variable, I excluded participants that did not reply to the question or provided implausible answers. | | | | | | |

1. **Analysis of Questionnaire Data**

| **Table C.1**: OLS regressions of participants’ belief that hard work brings economic success | | | |
| --- | --- | --- | --- |
|  | (1) | (2) | (3) |
| *Support for Redistribution* | -0.554*** | -0.558*** | -0.546*** |
|  | (0.040) | (0.040) | (0.042) |
| *Income* |  | 0.029 | 0.049 |
|  |  | (0.036) | (0.040) |
| Constant | -0.000 | -0.169 | 0.096 |
|  | (0.033) | (0.230) | (0.328) |
| Control for gender, age & field | No | No | Yes |
| Control for sessions | No | No | Yes |
| Observations | 380 | 371 | 371 |
| R-squared | 0.334 | 0.344 | 0.351 |
| *Notes:* The dependent variable is participants’ belief that hark word determines economic success in general*.* For this variable, I use a factor analysis on answers to questions about participants’ views about real-life determinants of economic success and create a univariate measure of the extent to which participants believe that it is due to hard work rather than luck. The questions asked are provided in Appendix B in Table B.2.  The variable *Support for Redistribution* refers to participants’ support for redistribution in general. For this variable, I use a factor analysis on answers to questions about participants’ redistributive attitudes toward redistribution and create a univariate measure of the extent to which participants support redistribution in real-life context. The questions asked are provided in Appendix B in Table B.3.  The variable *Income* refers to (ln) participants’ disposable income. The question is: “How much money (CHF) do you have at your disposal each month (approximately, after housing costs)?”  OLS estimates are reported, with *t* statistics in parentheses (* significant at 10 percent; ** significant at 5 percent; *** significant at 1 percent). | | | |

This section examines the relationship between participants’ views regarding real-life determinants of economic success and attitudes toward redistribution using the questionnaire of this study. In Table C.1, I regress participants’ belief that hard work brings success against their support for redistribution. The first column reveals a negative and significant relationship between both variables. This suggests that the less participants support redistribution, the more they think economic success is due to hard work. This is also true if I control for income (second column). The coefficient for *Income* is positive but not statistically significant, indicating if anything that the richer participants are, the more they think economic success is due to hard work. The third column controls for gender, age and field of study, and for session fixed effects and confirms previous findings. The significant and robust negative relationship between redistribution and beliefs about the sources of income is consistent with findings using observational data described in Section 3.

1. **Post-Experiment Questions about the Experiment**

| **Table D.1**: Description of the post-experiment questions | |
| --- | --- |
|  | Description |
| *Question 1* | Beliefs about the probability that their Work Performance is in the top half at the session level (*incentivized**). |
| *Question 2* | Belief about the probability that the Work Task determined the earnings reported by the high-income participants on average (*incentivized**). |
| *Question 3* | Willingness to know the probability the Work Task determined earnings that the computer assigned to their pair (Yes/No) |
| *Question 4* | State of the transfer they would have liked to implement if subjects reply “Yes” to the previous question and if they received a high income. |
| *Question 5* | Rating of the importance of beliefs in transfer decisions, from 0 (not at all important) to 4 (extremely important). |
| ** Questions 1 and 2 are incentivized using the same “matching probabilities” method used for eliciting the beliefs in Part II.* *Specifically, they have the highest chance to earn CHF 4 if they report their true beliefs.* | |

Analysis of the results

*Question 1*: When asked to guess the probability that their Work Performance ranks in the top half for the session, high-income (resp. low-income) participants reported in average a probability of 74.5 (resp. 62.7) in No Information and 74.1 (resp. 66) in Information. This means that participants reported a probability higher than 50 percent irrespectively on whether they received a high or low income. Using a two-tailed t-test, I find that the mean beliefs reported by high-income and low-income participants in both treatments are statistically different from 50—specifically, p<0.0001 in all cases. This suggests that both high- and low-income participants believe that they have a higher chance to win the Work Task. This is consistent with the idea that people are overconfident, i.e., they think they are better than average.

*Question 2*: When asked to guess the probability reported by those who received a high income on average, high-income (resp. low-income) participants reported a probability of 60.6 (resp. 58.6) in No *Information* and 61.4 (resp. 58.6) in *Information*. Using a two-sided t-test, the difference in means between treatments is not statistically significant neither for high-income participants (t(188)=-0.44, p=0.66) nor for low-income participants (t(188)=-0.03, p=0.98). This suggests that participants do not believe that those who received a high income exaggerate the role of work to justify giving less. Note that both high and low-income participants believed that high-income participants are more likely to attribute their income to work than luck which is accurate.

*Question 3*: When asked whether they would like to know the probability that Work Task determine the earnings in their pair, almost all participants reply yes.^[[1]](#footnote-1)^ This suggests that people do not want to hold wrong beliefs about the source of income and thus deservingness. Even though they do not distort their beliefs self-servingly to justify supporting a favorable redistributive policy, they still have wrong beliefs about their probability to perform better than the average.^[[2]](#footnote-2)^

*Question 4*: Importantly, when given this information, some of them declare to be willing to act accordingly. For those who wanted this information and who received a high income, I asked them whether they would have liked to implement another transfer. Among the 188 of them, 103 overestimated this probability and 83 underestimated it (thus 2 have correct beliefs). Among those who overestimated it, about one third (34%) declare they would have given more if they would have known the right probability and about two third (64%) state they would not have revised it (so 2% would have given less). Among those who underestimated it, 20% declare they would have given less if they would have known the right probability and 76% state they would not have revised it (so 4% would have given more). This suggests that some people are willing to change their redistributive decision when provided with accurate information. Even though any interpretation of these hypothetical answers must be done very cautiously, we could think that it is encouraging as it suggests that we could reduce the gap of the demand for redistribution between high-income and low-income individuals by providing them more accurate information about the role of work versus luck in income determination.

*Question 5*: Finally, I ask participants to rate the importance of beliefs in the decision to redistribute. Participants, whether they received a high or low income, think beliefs about the source of income are of average importance. Consistent with previous findings that beliefs are taken into account into the decision whether to redistribute, participants declare that, on average, beliefs are of average importance.

1. **Procedure**

| **Figure E.1**: Timing of the experiment |
| --- |
| 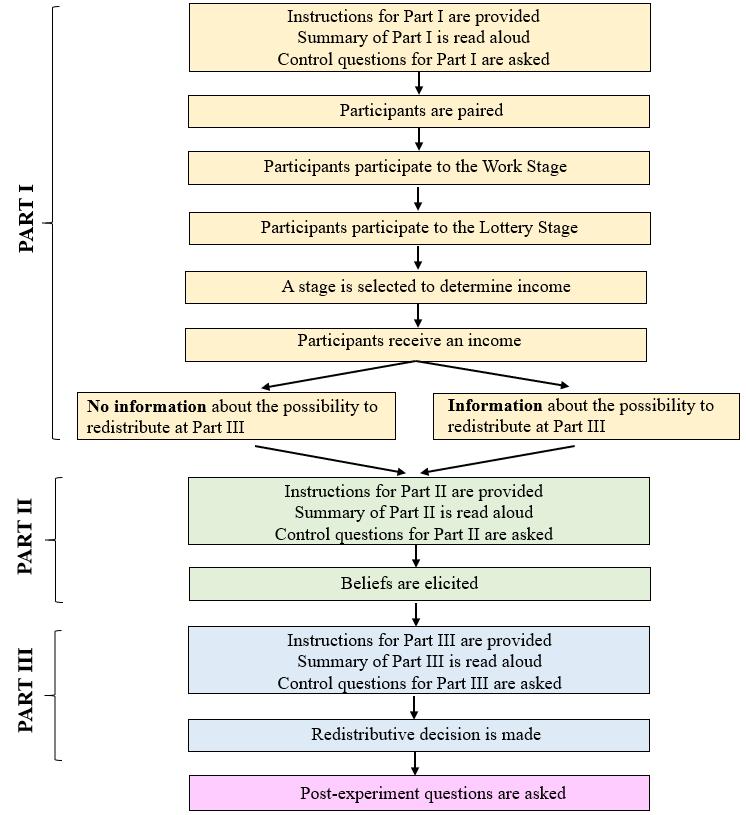 |

1. **Proofs**

**Proof A**

In the model presented in Section 5, I replace the assumption that the high-income agent considers it fair not to redistribute when she believes that work determines income (that is, $t_{i}^{F}=\left( 1-\overline{p}_{i} \right)\frac{1}{2}I_{P}+\overline{p}_{i}0=\left( 1-\overline{p}_{i} \right)\frac{1}{2}I_{P}$) with the assumption that she thinks low-income agent deserves a lower share, $\alpha<\frac{1}{2},$ when she believes that work determines income: $t_{i}^{F}=\left( 1-\overline{p}_{i} \right)\frac{1}{2}I_{P}+\overline{p}_{i}\alpha I_{P}$. Note that I continue to assume that a high-income agent would share equally income when income is certainly due to luck. This is commonly assumed in the literature (for example, Konow, 1996, 2000; Cappelen et al., 2007, 2010).

Under *No Information*, agents are not informed about the possibility to redistribute when forming their beliefs. This means that their beliefs are not influenced by transfers. Therefore, the hypothesis H1 remains the same. However, the transfer made by a high-income agent to a low-income one is now written as $t\left( {\overline{p}_{i}}^{NI} \right)=I_{P}[\frac{1}{2}(1-{\overline{p}_{i}}^{NI})-\frac{\gamma_{i}}{\beta_{i}}]+{\overline{p}_{i}}^{NI}\alpha I_{P}$. It follows that ${dt\left( {\overline{p}_{i}}^{NI} \right)}/{d{\overline{p}_{i}}^{NI}<0}$ since $\alpha<\frac{1}{2}$. This means that hypothesis H2 holds.

Under *Information*, agents are informed about the possibility to redistribute subsequently when forming their beliefs. The high-income agent’s utility is now written such that:

$$U^{I}\left( \overline{p}_{i} \right)=\gamma_{i}\left[ 1-\frac{1}{2} \left( 1-\overline{p}_{i} \right)-\overline{p}_{i}\alpha+\frac{\gamma_{i}}{\beta_{i}} \right]I_{P}-\frac{{\gamma_{i}}^{2}}{2\beta_{i}}I_{P}-\mu\frac{{{{(\overline{p}}_{i}}^{NI}-\overline{p}_{i})}^{2}}{2}$$

The beliefs they hold under *Information*, ${\overline{p}_{i}}^{I}$, are given by the first-order condition such that:${\overline{p}_{i}}^{I}={\overline{p}_{i}}^{NI}+\frac{\gamma_{i}}{\mu}I_{P}(\frac{1}{2}-\alpha)$. It follows that ${\overline{p}_{i}}^{I}>{\overline{p}_{i}}^{NI}$ since $\alpha<\frac{1}{2}$. This means that hypothesis H3a holds as well. Since ${dt\left( {\overline{p}_{i}}^{NI} \right)}/{d{\overline{p}_{i}}^{NI}<0}$ when $\alpha<\frac{1}{2}$, then $t\left( {\overline{p}_{i}}^{I} \right)<t\left( {\overline{p}_{i}}^{NI} \right).$ The hypothesis H3b also holds in this case.

Intuitively, all my results hold because the share given to low-income agents when income is due to work is lower that the share given when income is due to luck (i.e., $\alpha<\frac{1}{2}$). For this reason, high-income agents still have an incentive to exaggerate the extent to which they believe income is due to work to justify giving a lower share.

**Proof B**

Let $p\left( \bar{I}/W \right)$ be a continuous random sample with range [0,1] and probability density function $f_{P}(p)$. We assume that the probability density function (PDF) is symmetric around 0.5. Therefore, we have $f_{P}\left( p \right)=f_{P}\left( 1-p \right)$. We can write the expectation as follows:

$${{E[\overline{p}}_{i}}^{NI}]=E[\frac{0.5*p_{i}(\bar{I}/W)}{0.5*p_{i}(\bar{I}/W)+0.25}]$$

$${{E[\overline{p}}_{i}}^{NI}]=\int_{0}^{1} \frac{0.5*p_{i}(\bar{I}/W)}{0.5*p_{i}(\bar{I}/W)+0.25} f_{P}\left( p \right)dp$$

$${{E[\overline{p}}_{i}}^{NI}]=\int_{0}^{1} \frac{0.5*x_{i}}{0.5*x_{i}+0.25} f_{P}\left( 1-x \right)dx$$

$${{E[\overline{p}}_{i}}^{NI}]=\int_{0}^{1} \frac{0.5*x_{i}}{0.5*x_{i}+0.25} f_{P}\left( x \right)dx$$

$${{E[\overline{p}}_{i}}^{NI}]=E[\frac{0.5*(1-p_{i}(\bar{I}/W))}{0.5*(1-p_{i}(\bar{I}/W))+0.25}]$$

$$=E[{\underline{p}_{i}}^{NI}]$$

1. **Additional Results**

| **Figure G.1**: Relationship between transfers and beliefs in *No Information* |
| --- |
| 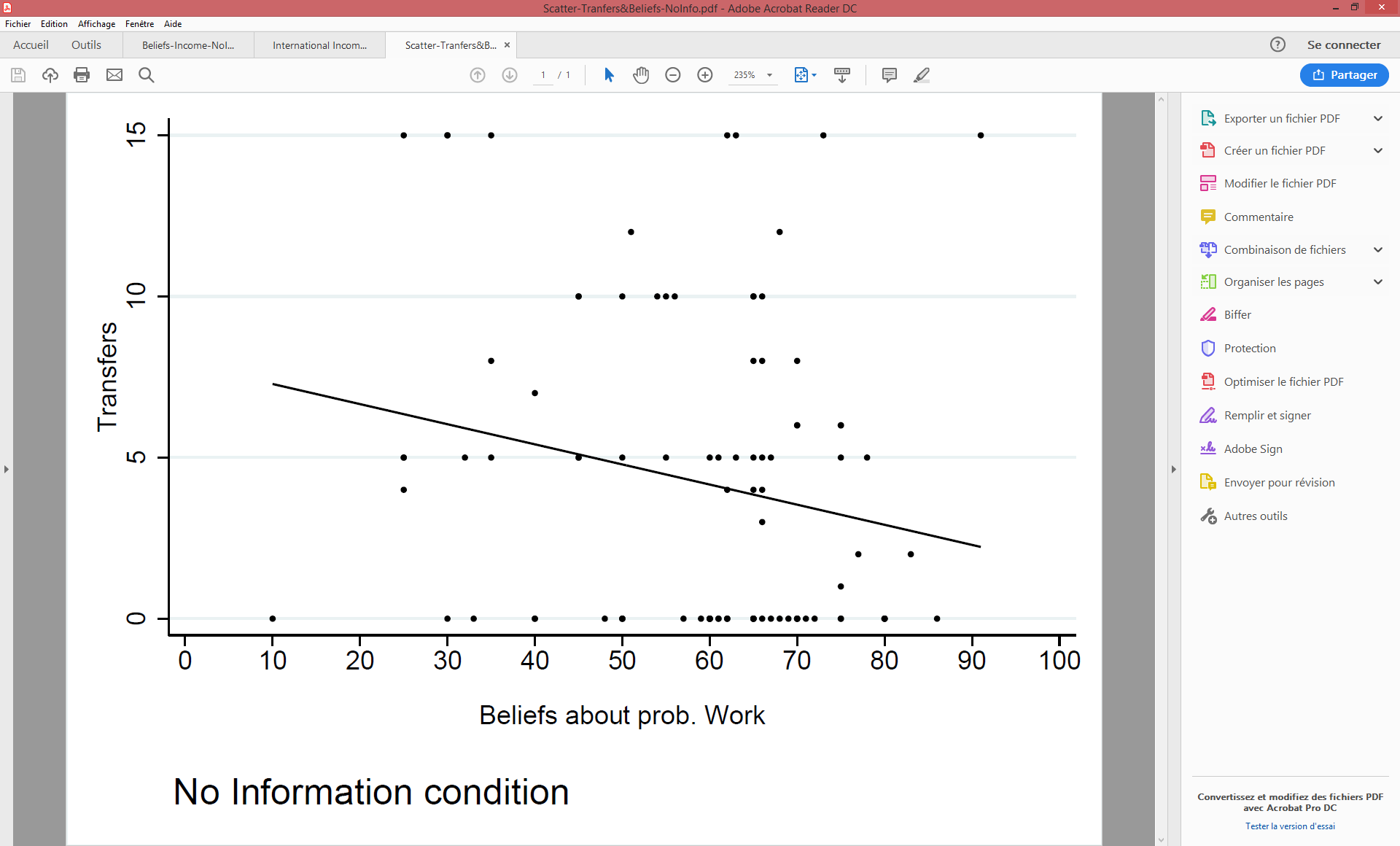  *Notes:* “Beliefs about Prob. Work” on the x-axis represent the elicited beliefs about the probability that the Work Task determined income rather than the Lottery. Each point in the scatter plot corresponds to a participant who received a high income in *No Information*. The solid line represents the fitted line from regressing transfers on beliefs; the coefficient being -0.062 (p-value=0.031). |

| **Figure G.2**: Average low-income participants’ beliefs about redistribution across treatments |
| --- |
| 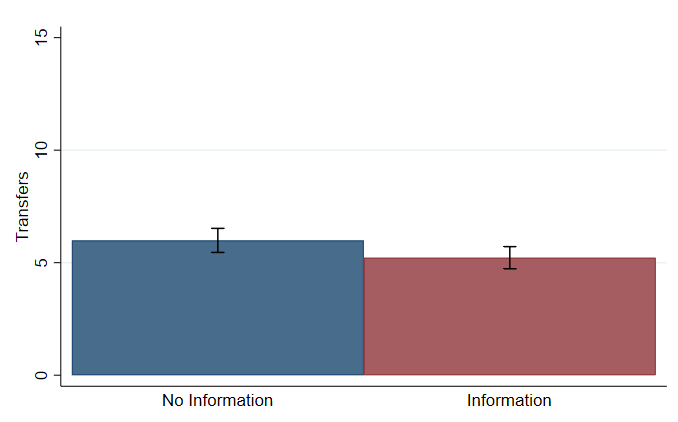  *Note:* “Transfers” on the y-axis represent the elicited beliefs about how much money low-income participants think that high-income participants will transfer. Error bars denote the standard error of the mean. |

| **Table G.1**: Relationship between Probability to transfer /amount transferred and *Information* treatment | | | | | | |
| --- | --- | --- | --- | --- | --- | --- |
|  | Probability to transfer | Amount transfered | Probability to transfer | Amount transfered | Probability to transfer | Amount transfered |
|  | (1) | (2) | (3) | (4) | (5) | (6) |
| *Information* | -0.159 | 0.114 | -0.095 | -0.812 | -0.069 | -0.812 |
|  | (0.183) | (0.985) | (0.212) | (1.233) | (0.183) | (1.205) |
| *Beliefs about probability of Work* |  |  |  |  | -0.015** | -0.039 |
|  |  |  |  |  | (0.006) | (0.036) |
| Constant | 0.119 | 7.243*** | 1.195 | 10.083*** | 2.043** | 11.899*** |
|  | (0.129) | (0.670) | (0.981) | (5.064) | (1.039) | (4.640) |
| Control for gender, age & field | No | No | Yes | Yes | Yes | Yes |
| Control for sessions | No | No | Yes | Yes | Yes | Yes |
| Observations | 190 | 98 | 190 | 98 | 190 | 98 |
| *Note:* Craggs double-hurdle estimates are reported, with standard errors in parentheses (* significant at 10 percent; ** significant at 5 percent; *** significant at 1 percent). Models (1), (3) and (5) are probit models, (2), (4) and (6) are truncated linear regressions (truncated from below CHF 0). | | | | | | |

1. **Experimental Instructions**

In the following, I provide the instructions for Part I, the instructions for Part II for the condition *Information* (the instructions for Part II for the condition No *Information* are similar but does not have the reminder of Part III at the end), and the instructions for Part III.

General instructions

Welcome to this study.

Please read the following instructions carefully. For participating in today’s study you will receive **CHF 10**. During the study you may earn **additional money**. The exact amount you receive will depend on your decisions and those of the other participants. Your final payment will be given to you in cash at the end of today’s study.

All of your interactions with other participants are **completely anonymous**. You will never learn the identity of the participants with whom you interact. They will also never learn your identity. You will not know which choices were made by a specific participant and no other participant will know which choices were made by you.

Communication with the other participants is strictly forbidden during the study. Violation of this rule will lead to exclusion from the study and loss of all payments.

This study will have **three parts**. We will explain **the exact procedures for Part I on the next pages**. Instructions for Parts II and III will be provided after you have completed Part I.

Instructions for Part I

At the beginning of Part I, you will be **randomly paired** with another participant. In the following, we will refer to the participant with whom you are paired as “*your paired participant.*” Note that your paired participant will **remain the same during the entire study** (i.e., for Parts I, II and III).

Part I will consist of two stages: a **Work Task** and a **Lottery**.

A. Work Task

In this stage, you will have the opportunity to work on a simple task. Specifically, you will be asked to count the number of times the number one (“1”) appears in a series of grids. The picture below shows an example of a grid you may solve by counting the number of ones:


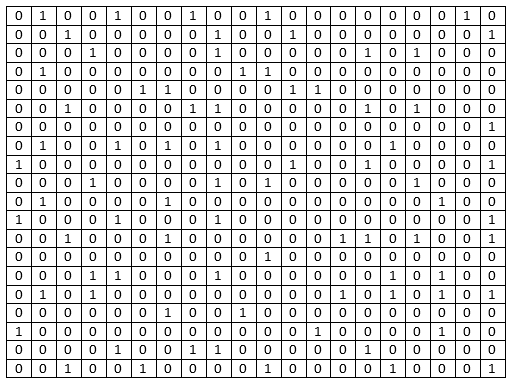


In the above example, the grid contains 78 ones. The correct answer is thus 78.

After you have entered your answer (i.e., the number of ones in the grid) and clicked the “OK” button, the computer will check your answer. If you enter the correct number the computer will count that grid as solved. If you enter an incorrect number the computer will not count that grid as solved. The computer will not tell you whether or not you solved the grid. In either case you will then see a new grid. All participants will be presented with the same grids.

Your goal is to solve **as many grids as possible.** The total number of grids solved represents your performance in this Work Task, namely your **Work Performance**.

You will have **20 minutes** to solve as many grids as you can. During the Work Task, you may decide to take a break from counting the number of ones, and thereby solving grids, and to stop working. However, the time will continue counting down while you take a break. If you decide to take a break please remain seated in front of your computer and keep quiet.

At the end of the Work Task, the computer will compare your Work Performance with the Work Performance of your paired participant**. Whoever has the higher Work Performance in the pair will be the winner of the Work Task.** That is, the person in your pair who **performs best** on the Work Task will be the winner. Note that **neither you nor your paired participant will know** who is the winner of the Work Task**.**

(*If both you and your paired participant have the same Work Performance the computer will randomly select one of you as the winner.*)

B. Lottery

In this stage, **luck will determine which participant in your pair** (i.e., you or your paired participant) **is the winner of a Lottery**. Essentially, the computer will flip a coin to select one of you as the winner. Each participant in a pair has the same chance to be selected as the winner of the Lottery. Note that **neither you nor your paired participant will know** who is the winner of the Lottery.

C. Earnings

After all participants have participated in the Work Task and the Lottery, the computer will **randomly select one of the two stages to determine each pair’s earnings**. This means that the earnings in your pair will be either the result of your and your paired participant’s Work Performance or luck in the Lottery. The winner of the selected stage will receive CHF 30 and the paired participant will receive CHF 0.

Specifically,

- If the computer selects the Work Task, then the winner of the Work Task will receive CHF 30 and the paired participant will receive CHF 0. *This means that earnings are entirely the result of which participant has a higher Work Performance.*
- If the computer selects the Lottery, then the winner of the Lottery will receive CHF 30 and the paired participant will receive CHF 0. *This means that earnings are entirely the result of which participant has more luck in the Lottery.*

At the beginning of Part I, the computer will **determine for each pair a percent chance that the Work Task,** rather than the Lottery, **will determine that pair’s earnings**. The percent chance that Work Performance, rather than luck in the Lottery, will determine the earnings in your pair is called *p*. A different percent chance is allocated to each pair, and *p* can take any value (integer) between 0 and 100, with each value being equally likely to be drawn.

At the end of Part I, the computer will use a random draw **based on *p*** to determine whether the Work Task, rather than the Lottery, will determine the earnings in your pair.

To better understand this idea of “percent chance”, let’s illustrate it with the example of balls in an urn. Imagine an urn containing 100 balls. Each ball may be either blue, in which case it corresponds to Work Performance, or it may be red, in which case it corresponds to luck in the Lottery. The computer will randomly select one ball from the urn and this ball’s color will determine whether the earnings in a pair will be based on Work Performance (blue) or luck in the Lottery (red).

Thus, the **number of blue balls** in an urn corresponds to the **percent chance** that the Work Task will determine the earnings in a pair, rather than the Lottery.

For example, if an urn contains:

- 0 blue balls (and thus 100 red balls), this means that there is a 0 percent chance (i.e., there is no chance) that Work Performance will determine the earnings in a pair. Thus, it is certain that the earnings in a pair will be determined by luck in the Lottery.
- 50 blue balls (and thus 50 red balls), this means that there is a 50 percent chance (i.e., it is equally likely) that Work Performance will determine the earnings in a pair. Thus, it is also equally likely that the earnings in a pair will be determined by luck in the Lottery.
- 100 blue balls (and thus 0 red balls), this means that there is a 100 percent chance (i.e., it is certain) that Work Performance will determine the earnings in a pair. Thus, there is no chance that the earnings in a pair will be determined by luck in the Lottery.

The actual number of blue and red balls can be anywhere between 0 and 100. Also, remember that each pair has its own designated urn containing a random number of blue (and thus red) balls. The computer will generate your pair’s urn at the beginning of Part I, and a ball will be randomly drawn from this urn at the end of Part I to determine your pair’s earnings.

**You will only be informed about your earnings and the earnings of your paired participant**. However, you will NOT know the percent chance that was assigned to your pair, nor will you know whether those earnings were based on Work Performance or luck in the Lottery. That is, you will not know the exact composition of your designated urn in terms of blue and red balls, nor the color of the ball randomly drawn.

Summary of Part I:

- The computer will randomly form pairs of participants.

- The computer will determine, for each pair, a percent chance, *p*, that Work Performance will determine that pair’s earnings. That is, the computer will generate an urn for each pair.

- All participants will participate in the Work Task. The person in the pair with the higher Work Performance will be the winner of the Work Task. Participants will not know who is the winner of the Work Task.

- All participants will participate in the Lottery. The computer will determine, by chance, one person in the pair to be the winner of the Lottery. Participants will not know who is the winner of the Lottery.

- The computer will use a random draw based on *p* to determine whether Work Performance, rather than luck in the Lottery, will determine the earnings in a pair. That is, a ball will be randomly drawn from each pair’s designated urn to determine that pair’s earnings.

- The winner of the selected stage (i.e., Work Task or Lottery) will receive CHF 30, and the other participant in the pair will receive CHF 0.

- Participants will only learn about their earnings and the earnings of their paired participant, not about the percent chance *p* that the computer assigned to their pair, nor the stage selected to determine their pair’s earnings. That is, participants will not know the exact composition of their designated urn in terms of blue and red balls, nor the color of the ball drawn.

If you have any questions, please raise your hand and wait for an experimenter.

Instructions for Part II

In Part II, you will be asked to **guess how likely it is that the earnings in your pair were determined by Work Performance** rather than by luck in the Lottery. That is, we ask you to guess the percent chance, namely *p*, that the earnings in your pair are entirely the result of your and your paired participant’s Work Performance.

Using the example of balls in an urn, we ask you to guess the number of blue balls in the urn that the computer generated for your pair. Recall that the number of blue balls corresponds to the chance that Work Performance, rather than luck in the Lottery, determined the earnings in your pair.

**To report your guess***,* you will **move a slider** along a bar representing the percent chance, *p*, which ranges from 0 (very left end of the slider) to 100 (very right end of the slider). As you adjust the slider to the percent chance *p* you believe is true, the number of blue balls in an urn of 100 balls on the screen will change to illustrate your choice of *p*. The picture below shows the slider you will be asked to move. Note that the position of the slider below (i.e., *50 percent chance, corresponding to 50 blue balls in the urn*) is just an example to illustrate the connection between the percent chance *p* and the number of blue balls in your designated urn.


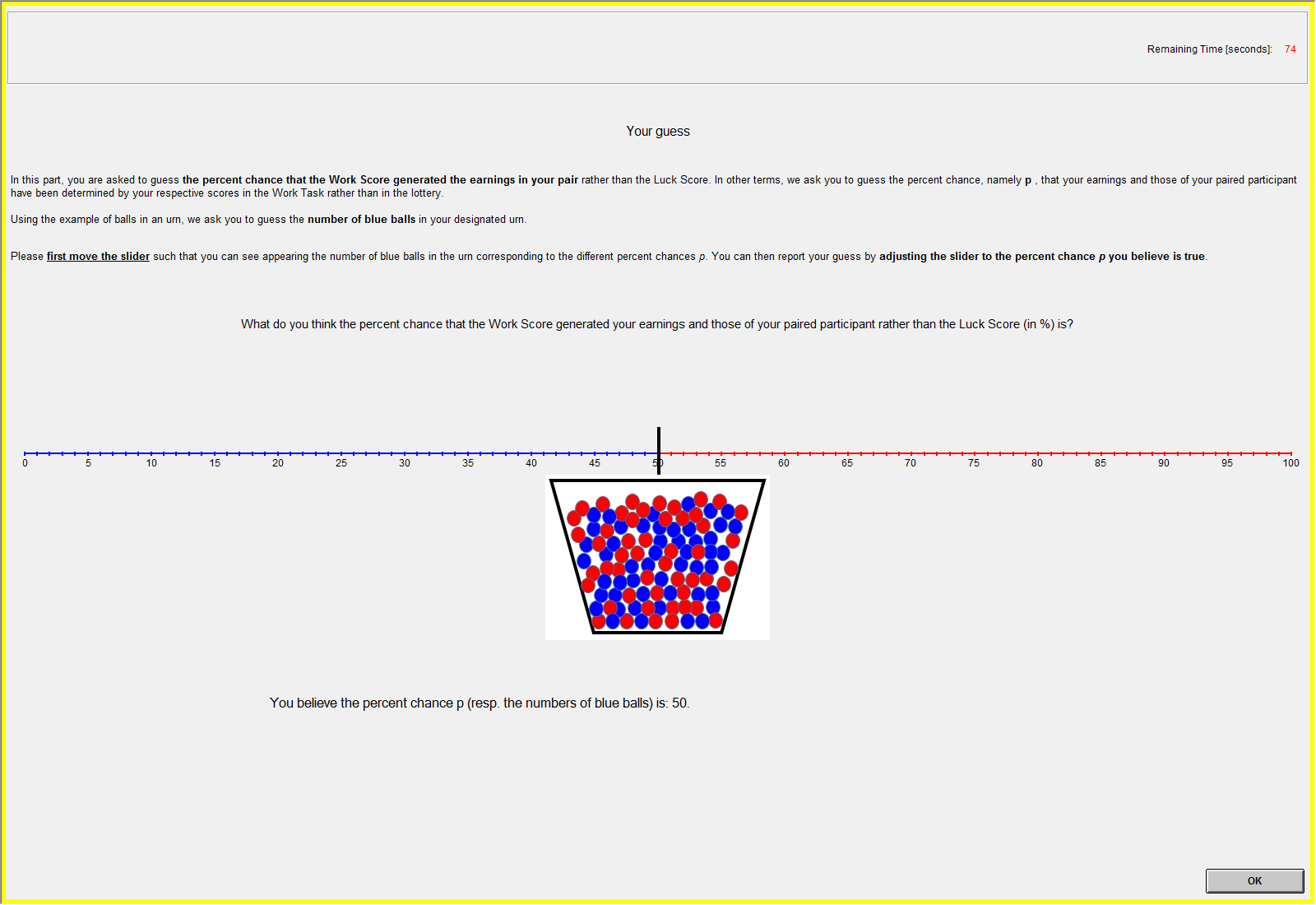


Your task in Part II is to position the slider to indicate how likely you believe that Work Performance, rather than luck in the Lottery, determined the earnings in your pair. This is the same as using the slider to create an image of the urn that you believe determined the earnings for your pair.

**Incentives to accurately report your best guess about the percent chance *p***

To give you an incentive to think carefully about the percent chance you believe that the earnings in your pair were determined by Task Performance, rather than luck in the Lottery, we introduce an “accuracy” payment rule that we explain in detail below.

Before explaining this rule, we point out a simple property it has: **you have the highest chance to earn money when you indicate exactly what you think is the percent chance *p* that the earnings in your pair were determined by Work Performance** (e.g., if you think there is 75 percent chance the earnings in your pair were determined by Work Performance, you should report a value of 75, and not something higher or lower, in order to earn the most money).

| We now explain how the accuracy payment rule works. Your best guess about *p* essentially determines on which of two urns you prefer to bet in order to win an additional CHF 4 payment. By making a guess of *p*, you indicate whether you prefer to bet either on your pair’s designated urn (which has an actual percent chance *p* of winning you CHF 4) or on a different urn with a percent chance *x* of winning you CHF 4.  At the end of Part II the computer will randomly draw a random number, *x*, between 0 and 100 (all the numbers are equally likely to be drawn). The computer will then compare *x* to the value you provided as your best guess of *p*.   - **If your best guess of *p* is higher than x**, then the computer will draw a ball **from your pair’s designated urn** and will pay you CHF 4 if the ball is blue and CHF 0 if the ball is red. - **If your best guess of *p* is equal to or lower than x**, then the computer will draw a ball **from an urn containing *x* blue balls** and (100 – *x*) red balls and will pay you CHF 4 if the ball is blue and CHF 0 if the ball is red.   This means that you should indicate, as your best guess of *p*, the exact number of blue balls that you believe your pair’s designated urn contains. If you indicate a higher number, then you may end up with your designated urn when you would have preferred having the urn with *x* blue balls. If you indicate a lower number, then you may end up with the urn with *x* blue balls when you would have preferred having your designated urn. |
| --- |

If this description seems complicated, remember that **you have the highest chance to earn money if you report the percent chance you truly believe was assigned to your pair**, i.e., how many blue balls you believe were in your designated urn.

You will be informed about whether or not you received the additional CHF 4 at the end of the experiment.

Summary of Part II:

- All participants will be asked to report their best guess about the percent chance that Work Performance, rather than luck in the Lottery, determined the earnings in their pair.

- All participants can expect to earn the most money by reporting the actual value of *p* they believe is true.

- All participants will learn whether they receive CHF 4 at the end of the experiment.

Instructions for Part III will be provided after Part II is completed. Remember that in Part III, the participant who received CHF 30 will have the opportunity to transfer some of these earnings to the paired participant who received CHF 0.

If you have any questions, please raise your hand and wait for an experimenter.

Instructions for Part III

Recall that, within your pair, one participant received CHF 30 and the other received CHF 0 in Part I.

In Part III, **the participant in the pair who received CHF 30 will have the opportunity to transfer some of these earnings to the paired participant who received CHF 0**. More specifically, the participant who received CHF 30 will choose an amount to transfer between CHF 0 and CHF 15. This amount will then be transferred to the participant in the pair who received CHF 0. The participant who received CHF 0 will not be able to choose a transfer or to influence the transfer chosen by the participant who received CHF 30.

To make his/her choice, the participant who received CHF 30 will see **16 possible transfers**, as shown in the picture below. He/she will select the one he/she would like to implement within the pair.


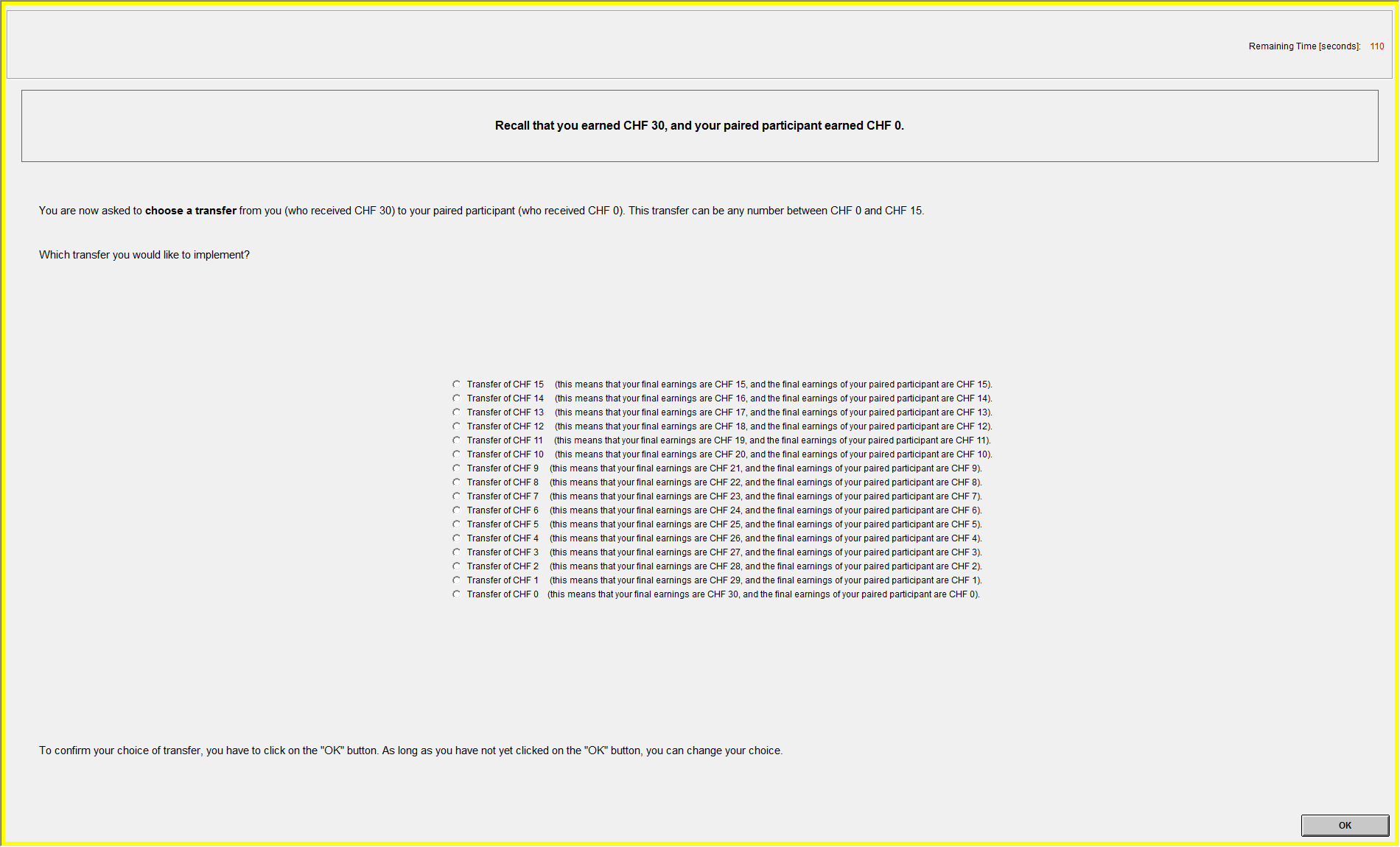


Note that for each possible transfer, we will also indicate the subsequent earnings for both paired participants.

**Your final earnings**

Your final earnings depend on your earnings in Part I and on the amount transferred in Part III by the participant who received CHF 30.

► If you received CHF 30 in Part I: Your final earnings = CHF 30 – amount transferred

► If you received CHF 0 in Part I: Your final earnings = CHF 0 + amount transferred

You will be informed about your final earnings, and whether or not you received the additional CHF 4 from Part II at the end of today’s study.

Summary of Part III:

The participant in the pair who received CHF 30 will have the opportunity to transfer some of these earnings to the paired participant who received CHF 0.

If you have any questions, please raise your hand and wait for an experimenter.

1. In *No Information*, about 98 percent of the high-income and 97 of the low-income participants want to know this probability, and 100 percent of the high-income and 98 of the low-income replied yes in *Information*. [↑](#footnote-ref-1)
2. This suggests that people do not consciously alter their perception of deservingness for self-esteem reasons, otherwise they would have probably avoided being informed about their actual control over their income. [↑](#footnote-ref-2)
